# Enhancement of Graphene-Coated Para-Aramid Yarn’s Pull-Out Resistance by Laser Pre-Treatment

**DOI:** 10.3390/ma19204379

**Published:** 2026-10-18

**Authors:** Silvija Kukle, Lyubomir Lazov, Rynno Lohmus, Ugis Briedis, Imants Adijans, Ieva Bake, Vladimir Dunchev, Erika Teirumnieka

**Affiliations:** 1 Institute of Architecture and Design, Riga Technical University, LV-1658 Riga, Latvia; silvija.kukle@rtu.lv (S.K.); ugis.briedis@rtu.lv (U.B.); 2 Rezekne Academy, Riga Technical University, LV-1658 Riga, Latvia; lyubomir.lazov@rtu.lv (L.L.); imants.adijans@rtu.lv (I.A.); erika.teirumnieka@rtu.lv (E.T.); 3 Institute of Physics, Faculty of Science and Technology, University of Tartu, 50090 Tartu, Estonia; rynnolo@ut.ee; 4 Department Material Science and Mechanics of Materials, Technical University of Gabrovo, 5300 Gabrovo, Bulgaria; v.dunchev@tugab.bg

**Keywords:** Kevlar^®^ KM2 and KM2+ fibre woven fabrics, ballistic application, laser pre-treatment, graphene coating, nano/microstructures, surface roughness, yarn pull-out force, continuous wave CO_2_ laser, fibre surface micro/nano-patterning

## Abstract

**Highlights:**

- Step I: Kevlar KM2 fibre fabric is irradiated with a low-power continuous wave CO_2_ laser beam; nano- and microstructures are formed on the surface of the fibres.
- Nanoscale bumps and shallow cavities, provided by an optimal set of laser parameters, increase the fibres’ contact surface area, significantly boosting the resistance to pulling yarns out of the fabric structure.
- Step II: With a para-aramid chemical structure, a compatible graphene-containing emulsion is synthesized by an ultrasonic-based technological process from graphite flakes; its zeta potential above 30 mV prevents aggregation and sedimentation of separated graphene sheets.
- Step III: During heat curing, as a result of nanosurface interactions at the molecular level, hydrogen bonds form between the active chemical groups on the para-aramid (KM2) fibre surface and the thin liquid layer of counter ions (the Stern layer) attached to the surface of the solid graphene sheets in the emulsion.
- In one graphene deposition process, the amount of graphene sheets applied to the fabric is limited, so the process needs to be repeated—normally three or more times—to achieve the desired effects.
- The experiments carried out show that the nanoscale topography of the surface created via laser pre-treatment with an optimal set of laser parameters provides better chances for more “encapsulated” graphene flakes to become anchored on the fibre surface, resulting in a synergistic increase in yarn pull-out junction rupture force (JRF).
- The experimental results under discussion show that the deposition of a two-layer graphene coating on laser-treated KM2 fabric fibres can give the same yarn pull-out JRF values as a three-layer coating on fabric that was not treated with a laser, which indicates the possibility of reducing the number of resource-intensive graphene deposition layers.

**Abstract:**

This study investigates the feasibility of enhancing graphene-coated para-aramid yarn’s resistance to pull-out from fabric structures, measured by junction rupture force (JRF), by increasing the fibre surface roughness with nano- and microstructures obtained by laser irradiation and graphene coating. Two types of Kevlar^®^ KM2 fibre woven fabrics were subjected to continuous wave CO_2_ laser processing to investigate the influence of laser power and scanning speed combinations on yarn pull-out force from the fabric structure under fixed laser spot diameter and raster step. Depending on the combination of applied laser parameters, a JRF increase of between 32% and 99% was achieved for laser-irradiated KM2+440 fabric compared to the JRF of untreated fabric. Applying a single layer of graphene coating to the fabric pretreated with optimal laser parameters doubled the JRF increase. When a second layer was applied, the increase tripled, indicating that cumulative effects were produced. As a result of laser treatment, the V2KM2600_laz variant’s JRF increased by 27% compared to the unmodified fabric, while the corresponding JRF difference for V3KM2600_laz was statistically insignificant, and any further JRF increase for V3 was only provided by the graphene coating layers. Meanwhile, for the laser-modified V2KM2600_laz, the JRF increase for the first graphene layer reached 56%, and adding a second graphene layer boosted it to 90%. The AFM and SEM imaging studies of the self-organization of nano- and microstructures on the surface of Kevlar^®^ KM2 fibres allowed us to conclude that irradiation-induced structures formed in the defect-enriched upper nanolayer of the 300–350 nm thick fibre skin, with a thickness of about 8 nm. Therefore, treatment within the examined laser parameter limits does not affect the fibres’ core structure.

## 1. Introduction

Aramid fibres (Afs) are widely used in various industries due to their exceptional strength, durability, and heat resistance, making them ideal for applications in the aerospace, automotive, military, and personal equipment sectors. Para-aramids (Kevlar^®^, Technora^®^, Twaron^®^) are also used for thermal protection, either alone or in blend forms, due to their 60% strength and modulus retention at 260 °C [1]. These fibres do not melt but char to a black colour. Aramids are resistant to many solvents and have low water absorbency but are sensitive to ultraviolet (UV). However, due to their chemical inertness, high crystallinity, and smooth surface, Afs often need surface modification to improve the chemical bonding and mechanical interlocking between the fibres and the matrix [2,3,4]. Surface modifications of Afs can improve surface chemical activity and increase surface roughness, aiming to achieve external chemical bonding and mechanical interlocking between the fibres and the matrix [5]. Various surface modification methods are employed to modify Afs, such as chemical grafting by attaching side chains or branches to a polymer backbone [6], physical methods, e.g., plasma treatment [7,8], Y-ray and UV irradiation [9], application of different coatings [10], and nanostructure construction on surfaces [11,12,13]. Oxygen plasma treatment for 5 min at 70 W remarkably increases the surface roughness of Kevlar^®^ fibre (KF) and provides an effective technique for overcoming the poor interfacial adhesion of KF-based bismaleimide composites [8]. Physical treatment methods like surface roughening and abrasion offer several advantages over chemical methods. They are typically more straightforward and more easily controlled, allowing for consistent modification without using specialized equipment or complex chemical reactions [14] that might damage the fibre’s inherent properties, making them a safer option for preserving the material’s integrity. However, while physical treatments can increase surface roughness and promote mechanical interlocking, they may not achieve the same level of strong interfacial adhesion as chemical methods [15]. Treatments such as surface roughening, plasma exposure, NaOH and silane coupling, and graphene nanoparticle (NP) incorporation, conducted to enhance Kevlar^®^ fabric–matrix bonding within thermoplastic polyurethane (K_TPU) and ethylene–vinyl acetate (K_EVA) matrices [16], showed the highest mean pull-out force from composites of untreated Kevlar^®^ samples. After roughening, the treated K_TPU samples displayed a lower mean pull-out force, with a 9.1% reduction, and the K_EVA samples showed a 26.6% reduction. These data highlight that, when thinking about plasma exposure, it is important to consider whether it might be the best choice for the specific use. Applying the combination of NaOH, silane, and graphene NP treatment yielded the highest adhesion, showing increases of 76.6% with TPU and 95.4% with EVA, underscoring the synergy between chemical coupling and nanomaterial reinforcement [16].

The use of laser systems for processing textile polymers Is increasing due to speed, accuracy and flexibility [17], as well as the emergence of new laser techniques for implementation. Laser-based methods enable high spatial resolution patterning of soft polymeric materials, providing targeted versatility and reliability without the need for stringent ambient conditions. In particular, the technique of laser-induced periodic surface structures (LIPSSs) has been successfully applied to the nano-structuring of polymer films using a polarized laser source at several laser wavelengths and pulse durations [18]. The possibilities of tuning the period and shape of the structures are discussed in this paper, since the control of the characteristics of the superficial structures can be crucial to meet the requirements of a specific application. Despite numerous successful modifications of metal surfaces and polymer films with LIPSS micro/nanostructures for surface functionalization, no publications apply this technology to the modification of woven or knitted polymer structures, which can be explained by the very different surface properties of these materials.

At the same time, laser surface texturing (LST) of textiles by applying existing engraving and marking technologies is a modern textile processing technique that offers precision, durability and desirable aesthetics at the macro level. It can be used for various applications, including fashion, home textiles, functional clothing, specific medical devices, furniture, filters, aviation and advertising. Laser marking and engraving are common applications; the main difference from cutting is the depth of processing. The laser beam induces only slight changes in appearance or colouration during laser marking. The marking itself is waterproof and extremely durable. Engraving can be done with the same equipment but with different process parameters [17]. Laser marking in textile technology replaces dry processes such as sandblasting, hand sanding, destroying, and grinding, which are the most environmentally polluting technologies. In addition, the technology is faster, more accurate and versatile [19]. While lasers have been widely used in other fields, their use in textiles for functional enhancements is a relatively new approach [20]. This technology addresses the need for environmentally safer materials by removing harmful chemical treatments, while its scalability ensures production viability for broader market applications [21]. LST micro/nano technologies are based on self-organized laser-irradiated structures and direct laser-inscribed structures. The possibilities are limitless for laser-inscribed structures, whereas for laser-irradiated structures, only two main types—random and periodic structures—have been reported [22]. Self-organized surface structures may consist of microstructures, nanostructures, or hybrid variants [23]. So far, there are few reports on the range of applications of laser-irradiated structures compared to the wide range of applications offered by laser-inscribed structures [24,25]. The possible technological constraints of laser processing are largely determined by the expected processing effects, the structure specifics of the material subjected to processing, and its diverse properties as a material [26]. Research activities involve surface design for different applications, modelling and setting up laser processing parameters, tailoring surface properties by laser irradiation, characterizing processed surfaces using both experimental and analytical methods, and testing engineered surfaces in proposed applications [27].

Carbon dioxide (CO_2_) lasers are suited for textile treatments because they provide targeted heating to a portion of the material surface, creating a photothermal effect. Depending on the wavelength, the penetration depth of most materials ranges from a few microns to hundreds of microns. Heat dissipation in a sample, which is affected by its thermal conductivity and the nearby environment adjacent to the irradiated region, forms a “heat-affected zone” [26]. In the photothermal process, the energy received from the laser beam increases the surface temperature of the material, leading to melting and/or vaporization. Photothermal processes induce surface modifications such as roughness in polymers. However, in the photochemical process, there is a direct breakage of molecules by highly energized photons incident on a material surface, hence inducing chemical modifications. The combination of both photothermal and photochemical processes modifies the roughness and chemistry of surfaces simultaneously [28].

**Figure 1:**
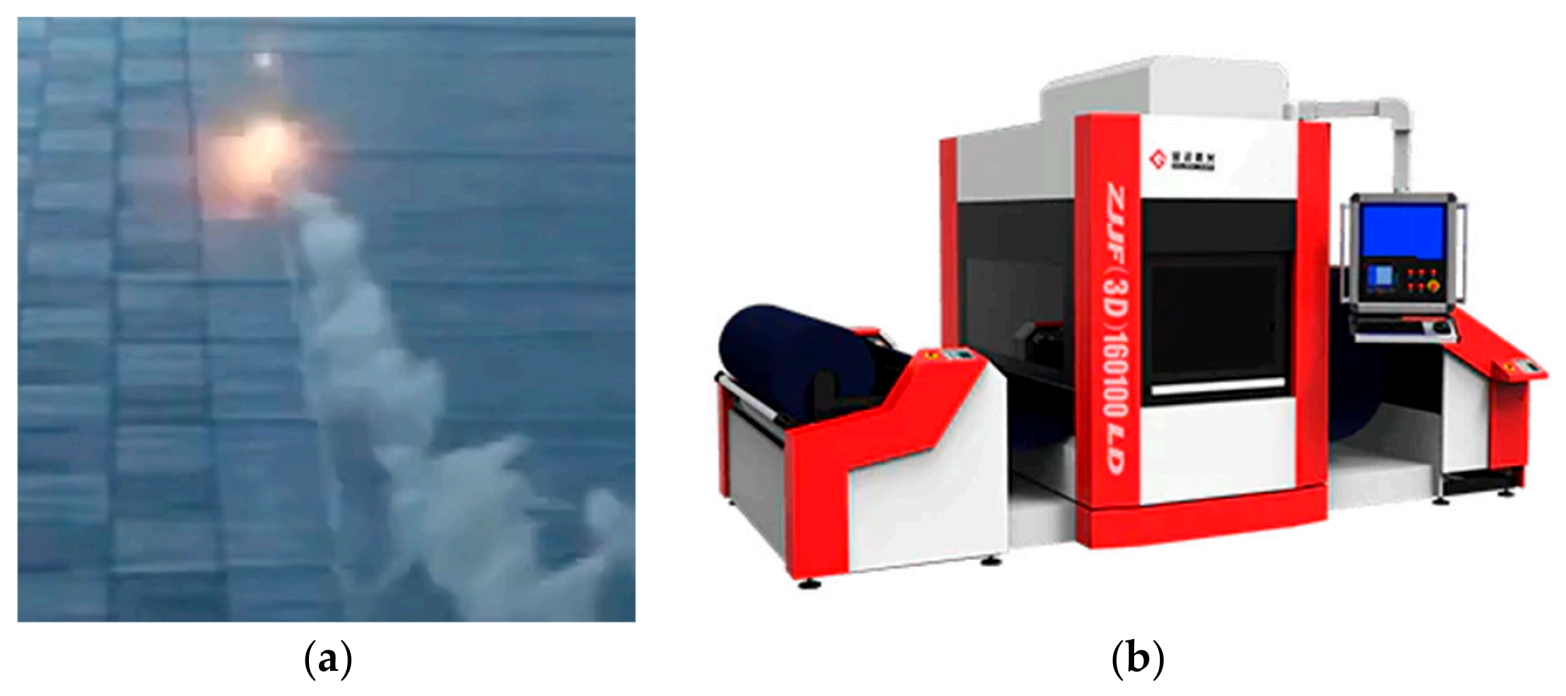
CO_2_ laser marking system for textiles continuous processing on the fly; (**a**) the 3D dynamic galvanometer system directed laser beam; (**b**) laser roll-to-roll engraving machine [30,31].

Laser marking on the fly refers to a marking technology that allows products to be marked while moving on a production line, without the need to stop the process. In this non-stop marking technology, the laser system synchronizes with the speed of the moving product [29]. Unlike traditional stationary marking, this method enables real-time engraving, coding or serial numbering while the product passes under the laser beam (Figure 1a). This ensures no slowdown in production speed, making it ideal for high-volume manufacturing. This advanced method ensures high-speed, high-accuracy identification and processing and can reach 1600 mm in width, supporting textile unwinding, modifying and rewinding.

The roll-to-roll laser treatment system (Figure 1b) was recently launched, equipped with an advanced CO_2_ laser that can create continuous effects on textile roll goods in a variety of finishing techniques, including cutting, engraving, perforating and marking, maintaining consistent quality through the entire roll. This technology opens new possibilities for modifying para-aramid woven, knitted, and non-woven textile surfaces with laser-irradiated macro-, micro- and nanostructures. To take advantage of these possibilities, it is necessary to determine the allowable limits for changes in laser processing parameters and to coordinate sets of parameters that would ensure or improve the required functional performance without damaging the structure of the textiles/fibres being modified, as well as to evaluate the effectiveness of the modifications qualitatively and quantitatively with indicators appropriate for the application.

Fabrics made from para-aramid fibres exhibit exceptional strength, high modulus, and superior ductility, making them highly suitable for ballistic applications despite their relatively high cost. Kevlar^®^ K49, KM2 and KM2+ are very high modulus, high toughness forms of Kevlar^®^ designed for improved ballistic fragmentation resistance and energy absorption capacity. The ballistic forms of Kevlar^®^, in particular, KM2 and KM2+, are distinguished by relatively large values for both yield strength and strain, which translates into a large value for toughness, or work per unit volume at failure [32,33,34]. Yarn pull-out can be an important energy absorption mechanism during the ballistic impact of woven Kevlar^®^ fabric.

This study investigates the feasibility of increasing graphene-coated para-aramid yarn pull-out resistance from fabric structures by applying fibre surface micro/nano patterning by laser pre-treatment. Two types of Kevlar^®^ KM2 and KM2+ fibre woven fabrics were subjected to continuous wave (CW) CO_2_ laser processing to investigate the influence on yarn pull-out resistance from the fabric structure. Yarn pull-out force was assessed using junction rupture force (JRF) under various combinations of laser parameters to enhance the surface functionality of Kevlar^®^ ballistic-grade fabric without compromising its fibre core properties.

## 2. Materials, Experimental Setup and Methodology

### 2.1. Laser Pre-Treatment Specifics: Investigation of Kevlar^®^ Fibres and Fabrics

The failure mode of an aramid/resin composite is generally interfacial debonding with some fibrillation of the fibre surface [35,36], relevant to the skin/core structure formed in the aramid fibre extrusion and coagulation process [37], which is different from a carbon fibre-reinforced composite [4]. The core region tends to possess a high degree of order and crystallinity, while the microstructure of the skin is generally less crystalline [38]. The molecular interactions in the skin region are weak, so microfibrils can be detached from the fibre surface [36]. The special skin/core structure of an aramid fibre makes the interfacial failure mechanisms highly intricate. The quantitative effects of the skin/core structure of an aramid fibre on its adhesion properties are still not fully investigated.

The inhomogeneity in aramid fibre and yarn properties and fabric structures [23] makes fabric laser machining difficult; the process can significantly affect these materials, leading to various forms of damage, such as fibre fibrillation, skin fragment pull-out and structure damage, as well as heat damage. Thus, laser processing of fabric involves numerous parameters, which need to be optimized to achieve the required outcomes. These parameters include applied laser power, repetition rate, scanning speed, and a wide range of material parameters [17]. The effect of these parameters on process behaviours includes heat-affected zones, machining depth and material removal rate. The commonly used one-factor variation at a time approach requires many runs and does not allow for the investigation of process variable interactions.

Power (P) and raster setup determine the energy delivered. Laser speed (LS), often referred to as scanning speed, determines the duration of laser exposure on any part’s surface and indicates how quickly the laser beam moves across the material surface during the LST process. LS influences the depth, quality, and overall appearance of the produced effects and also impacts several facets of laser processing, including precision, efficiency, and material integrity. At lower speeds, the laser beam dwells longer on each point of the material, delivering more energy to the area. This increased exposure results in deeper and more intense marks, requiring significant material removal or high contrast, but it may also lead to excessive heating and potential material damage. This can cause unwanted effects such as melting or charring, particularly in heat-sensitive materials like polymers.

Higher LSs reduce the time spent on any one point, leading to shallower and less intense marks. This is suitable for surface-treated applications where only a slight alteration of the material is desired, such as light etching or marking coated surfaces. Polymers are more sensitive to heat and can benefit from higher speeds to prevent melting or evaporation. Fast LSs are more likely to have minimal thermal impact and produce cleaner marks. When marking depths of 0.127 mm or deeper are required, making multiple passes with high-speed settings can be more efficient than marking at lower speed settings. However, if the LSs are set too high, the laser might not generate enough heat to produce the necessary effects. When the laser beam contacts the surface of the material, its concentrated energy causes controlled vaporization in the case of aramid. Depending on the settings—power, speed, and raster setup—this produces either subtle etchings or profound engravings.

Even if these three main CO_2_ laser parameters are coordinated to ensure the planned intensity of fibre surface modification, local heat damage may occur due to structural irregularities and defects on the fabric surface. The most common heat damage from varying intensity affects fibres raised on the fabric surface, especially when defects resulting from technological processes have accumulated there [23].

**Figure 2:**
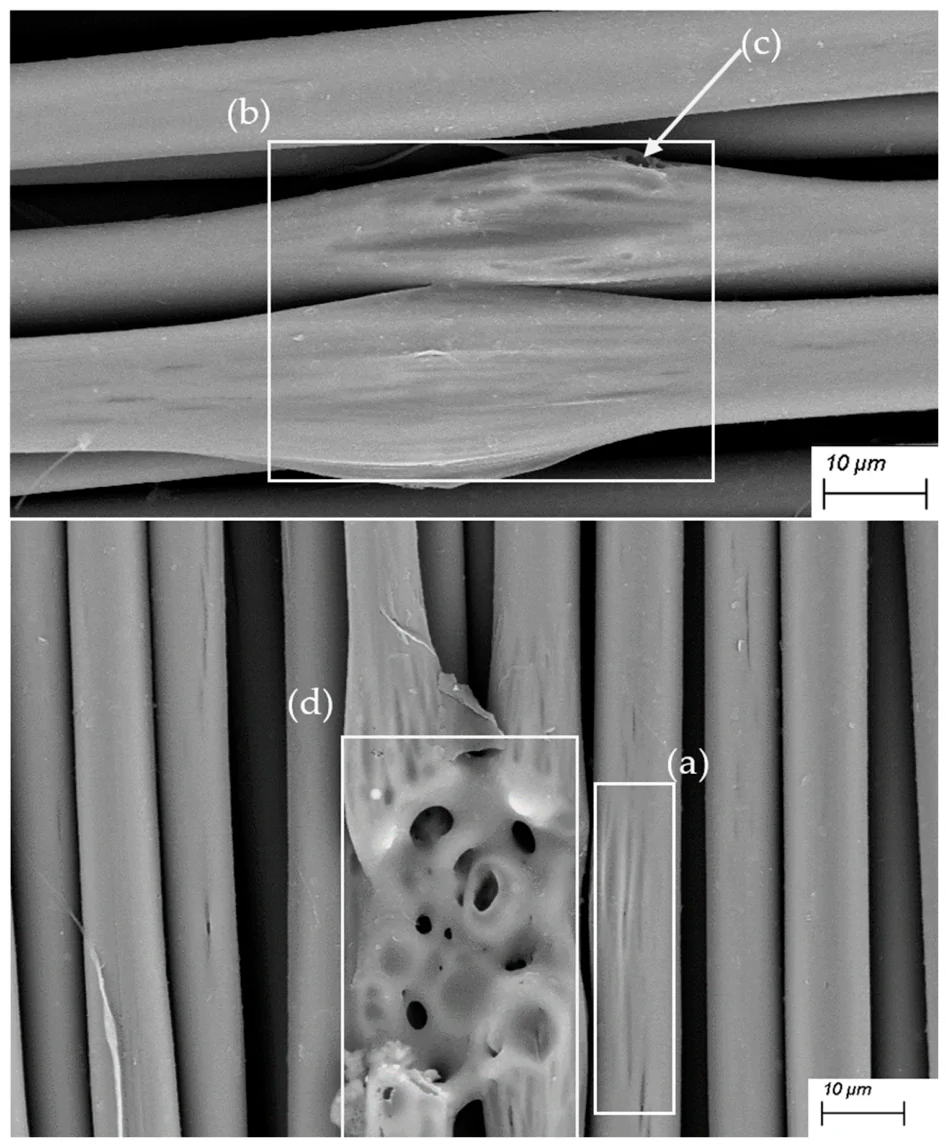
Local heat damage of KM2600_laz fabric fibres. (**a**) Micrometre-sized surface corrugations; (**b**) increased diameter of affected fibres; (**c**) initial cracks in the fibre skin; (**d**) deep structural damage of the fibre internal core. Mag., × 1000.

Scanning electron microscope (SEM) micrographs obtained within the framework of this study (Figure 2) show that as the heat of the laser beam increases, micrometre-sized surface corrugations produced on Kevlar^®^ fibre surfaces by laser ablation (Figure 2a) can locally increase the diameter of the affected fibres (more common defect) (Figure 2b). This indicates that under increased heat, evaporation of the aramid fibre core occurs, and volatile compounds accumulate under the skin. As the volume of accumulated volatile components increases, cracks initially appear in the skin (Figure 2c); the fibre diameter further increases until the skin completely decomposes, revealing deep internal core structural damage caused by evaporation (Figure 2b,d). In most cases, the damage affects two or even several adjacent fibre areas. Such defects are visually noticeable because they cause changes in colour due to carbonization.

**Figure 3:**
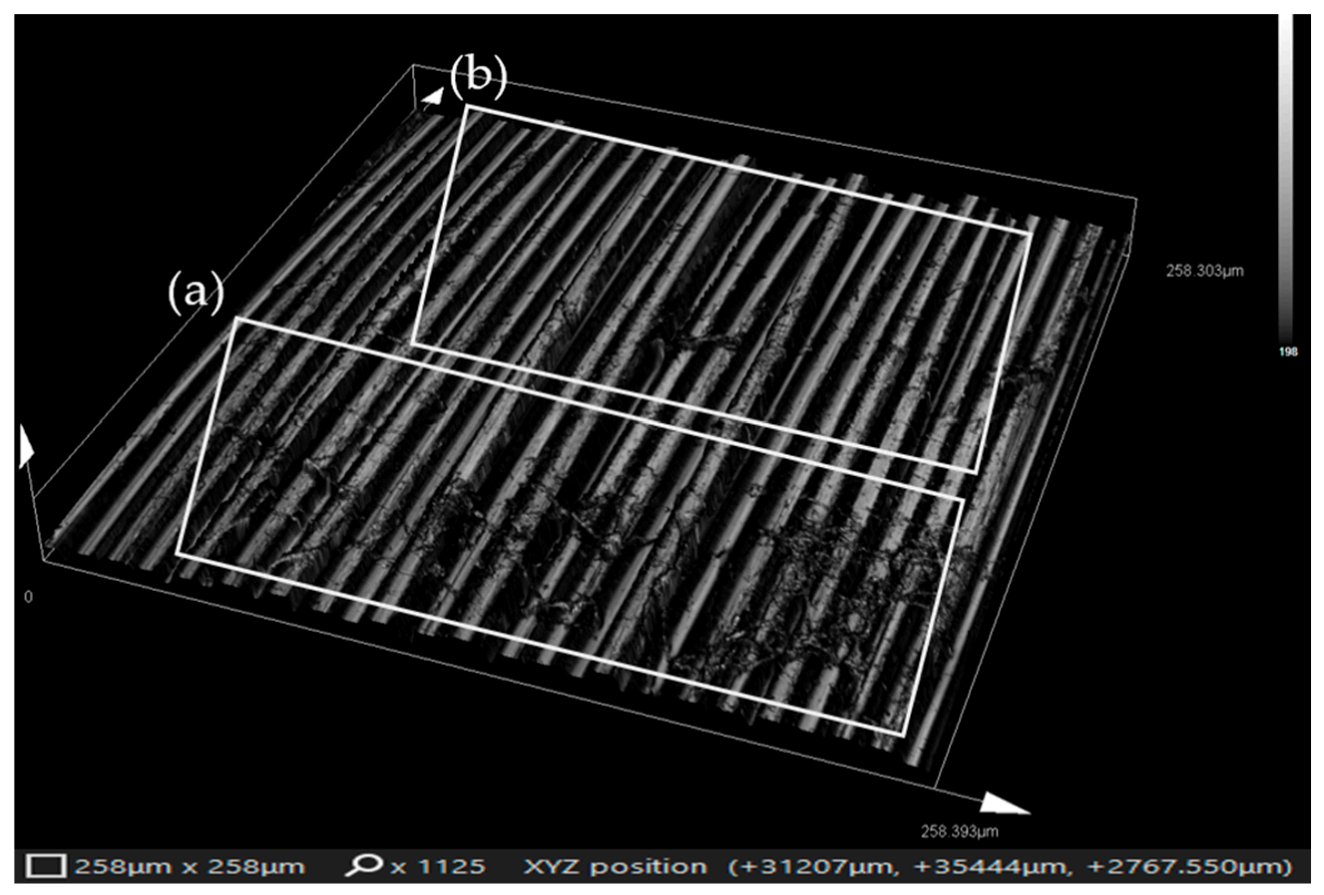
Confocal 3D image of as-received KM2600 fabric. (**a**) Areas with high surface contamination combined with the local elevation of individual fibres; (**b**) less contaminated surfaces after laser processing. Mag., ×1125.

Thus, the surface destruction of outer fibres begins beneath the fibre skin, with gaseous compounds accumulating and causing local attacks on the fibre skin. Cracks in the skin appear in directions of lower resistance, which, upon reaching a threshold, can result in the disruption of the fibre architecture, forming porous, thin-walled structures with pore sizes ranging from the nanometre level to several tens of micrometres (Figure 2d). If the laser parameters are not adjusted, the degradation process continues, leading to the complete evaporation of fragments in the overheated fibre groups [23]. Intensive or less intensive damage can be visually observed by the intensity of colour changes in the fabric. Fibre groups exposed to local damage rise above the fabric plane at yarn intersections, as well as in areas where defects accumulate (Figure 3a such as fibrillated fibres, pieces of skin fragments torn off in previous processings, clusters of applied finishes, etc. Areas with high surface contamination, combined with the local elevation of individual fibres, or more often fibre groups, above the fabric plane, are more susceptible to local fibre structure damage (Figure 3a). Laser processing of less contaminated surfaces (Figure 3b) with the same set of processing parameters occurs without causing deep damage to the fibre structure, as shown in Figure 2d. The authors’ experience indicates that after laser processing with the experimentally applied laser parameter sets, the number of defects created by previous technologies is significantly reduced.

**Figure 4:**
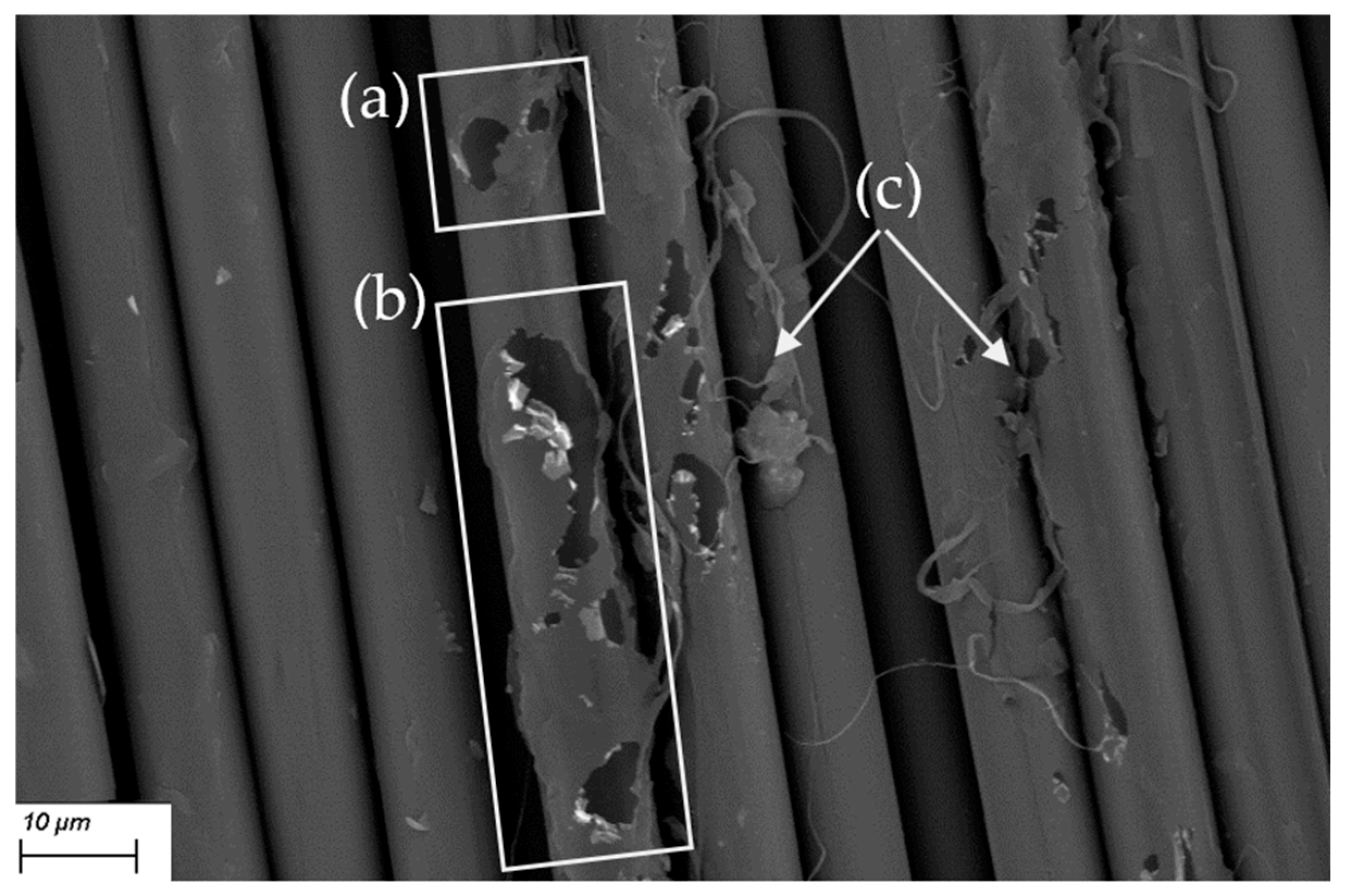
SEM image showing damage to a skin of fibres that rise above the KM2600 fabric plane caused by a laser working head. (**a**) Local damage to the surface of the fibre skin; (**b**) group damage to the surface of the fibres that rise above the plane of the fabric fibre; (**c**) loosely bound contaminants on the fibre surface that formed during previous technological and sample purification processes. Mag., ×1000.

The damage to the outer fibre skin shown in Figure 4 is less common and occurs when the laser working head touches the fibres that have risen above the plane of the fabric.

The micrographs in Figure 2, Figure 3 and Figure 4 illustrate the process of defect formation and its causes. Using optimized laser parameter sets, such defects should not occur. But when performing laser processing over large areas, local damage can occur in spots with high concentrations of surface defects that accumulated in previous technological processes. When developing technological documentation, acceptable limits according to the product range should be determined in the scale-up process by assessing the condition of the fabric surface to be treated.

### 2.2. Quasi-Static Yarn Pull-Out Test Methodology

**Figure 5:**
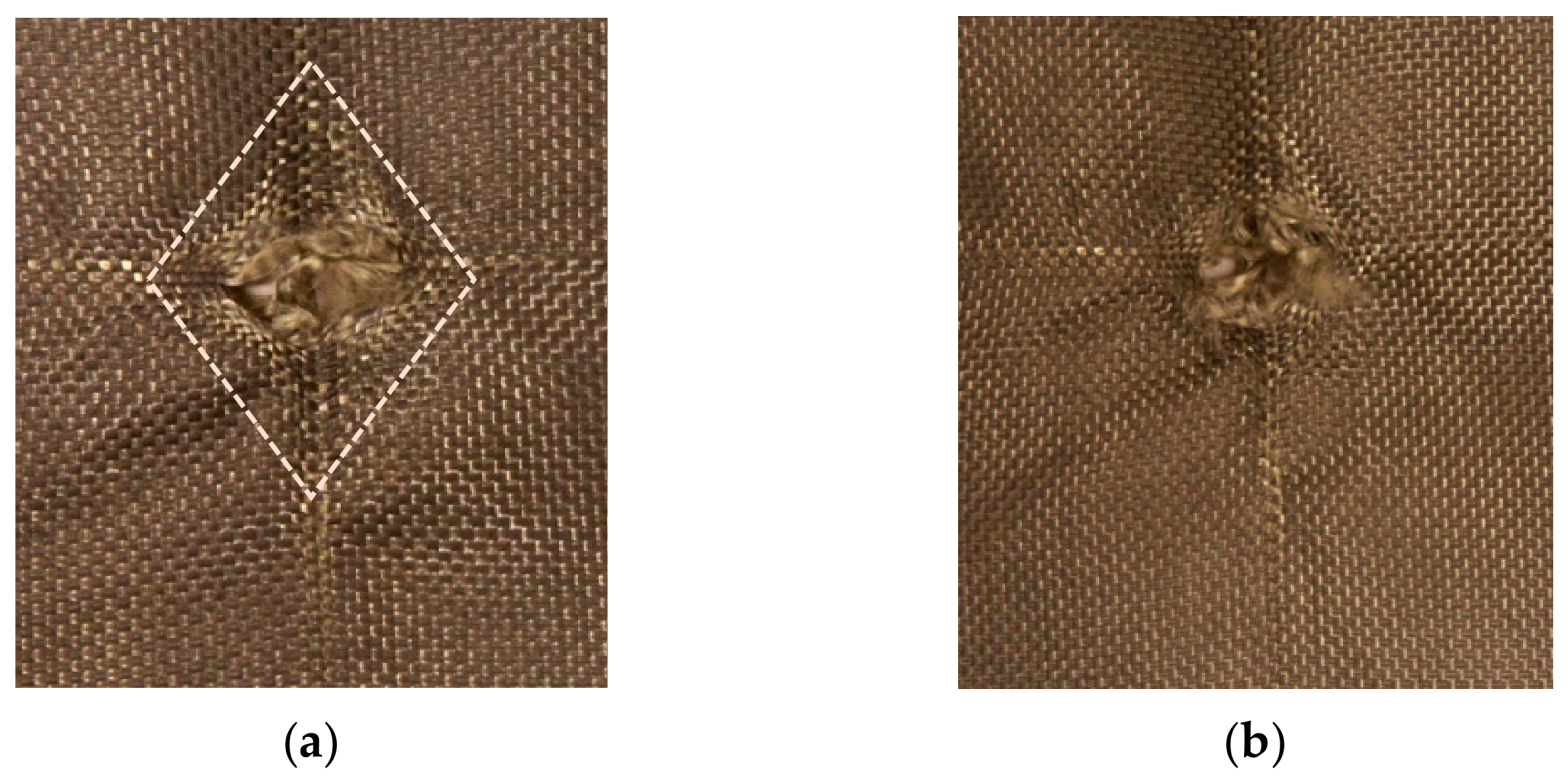
First graphene-functionalized KM2600 fabric layer of the soft non-perforated ballistic package, which gets perforated by a bullet. Striking face (**a**) and exit face (**b**) [40].

High-performance woven Kevlar^®^ fabrics are widely used in the military, where these structures are commonly exposed to impact loading, including bullets, fragments and blast waves. Yarn interaction is one of the key factors affecting the impact resistance of fabrics. Taking into account the critical role of yarn interactions in fabric performance, extensive research over several decades has been devoted to understanding the mechanisms of yarn pull-out behaviour. Dong et al. tested and modelled yarn pull-out in plain woven Kevlar^®^ fabrics and found a significant positive correlation between ballistic resistance in impact tests and yarn pull-out force [39]. A macroscopic study on the fabric structure failure mechanism of a Kevlar^®^ KM2600 graphene-functionalized plain weave fabric under ballistic impact conditions, as shown in Figure 5, shows that in the first layer of the soft ballistic package, the bullet’s impact energy is already dissipated by compacting threads and pulling yarns out of the fabric structure without breaking them.

As a result of experimental investigations of the role of frictional yarn pull-out on the impact response of Kevlar^®^ fabrics, some authors suggested that frictional sliding and pull-out of yarns serve as the dominant energy dissipation mechanisms under ballistic impact conditions [39,41]. Extensive research was done by the authors of [42] to understand the yarn pull-out behaviour under various loading conditions as a mechanism for dissipating ballistic impact energy in Kevlar^®^ KM2 fabric, allowing them to establish a quantitative relationship between quasi-static (QS) yarn pull-out behaviour and impact responses.

**Figure 6:**
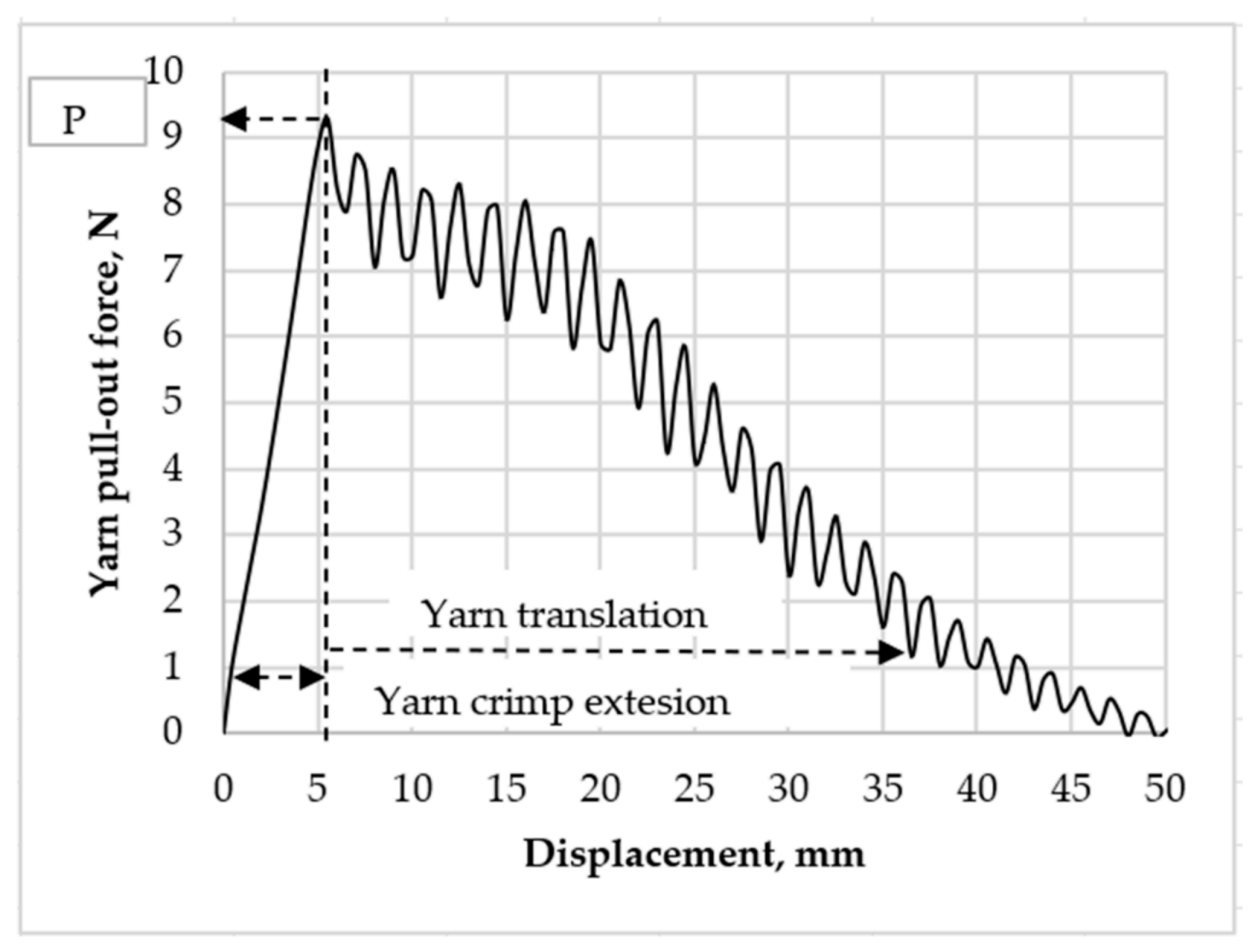
Yarn pull-out force–displacement curve of sample KM2600_2k.

To evaluate yarn pull-out resistance in this research, two fabric samples were prepared for each variant, with a size of 220 mm in the warp direction and 110 mm in the weft direction. In total, 20 pull-out test data for each variant in the form of yarn pull-out force–displacement curves were obtained. According to the methodology in [43,44], warp threads were unrolled from the bottom of the sample to a width of 10 mm and from the top to a width of approximately 60 mm, so that the height of the tested sample remained 50 mm. Yarn pull-out force measurements were obtained using an Instron Force transducer device, model 2519-107 (capacity: 5000 N, model 2519-107, ID: 3345K6537, Illinois Tool Works Inc., Norwood, MA, USA), and results were observed with the program Instron Bluehill Lite Version 2.17, (SN: 717479C, Illinois Tool Works Inc., Norwood, MA, USA) [23]. A pre-tension of 100 N and a pull-out speed of 50 mm/min were used. The peak pull-out force P_m_ of yarn, which is labelled as junction rupture force (JRF), was used to compare experimental variants (Figure 6). Tensile strain was measured synchronously with the yarn pull-out force at P_max_.

### 2.3. Materials

Two types of commercial ballistic fabrics woven using 600 and 440 denier yarns of Kevlar^®^ KM2 and Kevlar^®^ KM2+ fibres, respectively, were used to produce experimental samples: (1) KM2600 (INFINITY COMPOSITES INC, Ashtabula, OH, USA), plain weave, 11 threads per cm in the warp and weft directions, fabric thickness 0.23 ± 0.02 mm, areal density 146 g/cm^2^, and final UV protection finishing, and (2) KM2+440 (producer SAATI S.p.A, Milano, Italy), loom-state (without final finishing), plain weave, 14 threads per cm in the warp and weft directions, fabric thickness 0.17 mm, and areal density 125 g/cm^2^. The fabric samples were first cleaned with acetone and 2-propanol, then rinsed with deionized water and left to dry at room temperature.

Both fabric types subjected to the laser pre-treatment were subsequently modified with one or two layers of graphene coating prepared following a previously developed method by the authors of “Technological sequence to obtain graphene-modified Kevlar^®^ fabric” [40,45].

### 2.4. Laser Pre-Treatment

Continuous Wave (CW) CO_2_ Laser SUNTOP Model ST-CC9060 (Suzhou Suntop Laser Technology Co, Ltd., Suzhou City, China), which produces a continuous beam of 10.6 µm wavelength, was used. The beam diameter in focus, d = 100 µm, and the focal length, F = 63.5 mm, were kept unchanged. For the variables used in this study, including laser beam scanning speed (v), power (P) and raster step (∆x), the allowable variation limits were determined based on previous experimental studies [23]. The sets of laser pre-treatment variable parameters for Kevlar^®^ KM2+440 fabric are based on the 2^2^ Factorial Experimental Design and corresponding variant designations shown in Table 1 and Table 2.

Considering the obtained results, the two most successful sets of laser parameters were applied to modify Kevlar^®^ KM2600 fabric; variant designations and corresponding parameter setups are shown in the bottom part of Table 2. In the authors’ previous studies, the intervals of laser power and scanning speed variations were determined at a fixed laser beam diameter and raster step without affecting the Kevlar^®^ fibre architecture [23].

### 2.5. Technology to Obtain Graphene Coating on Laser Pre-Treated Fabric Samples

The method and technological sequence for obtaining graphene-modified Kevlar^®^ fabric developed by the authors of [40,45,46] were used to produce graphene-modified samples using laser pre-treated Kevlar^®^ KM2+440 and Kevlar^®^ KM2600 woven fabrics. The sample processing plans are shown in Table 3.

### 2.6. Surface Morphology Investigation

The equipment used to obtain and evaluate surface images, graphs and roughness included the following: (1) confocal laser scanning microscope OLYMPUS LEXT 3D MEASURING LASER MICROSCOPE OLS5000 model “OLS5100-EAF” (Olympus Corporation, Tokyo, Japan); (2) Atomic Force Microscope Dimension Edge (Veeco) (Billerica, MA, US, located in the Institute of Physics at the University of Tartu, Estonia); (3) SEM ZEISS (Oberkochen, Baden-Württemberg, Germany, located in the Vasil Levski National Military University, Bulgaria). Gold sputter coatings were applied to prevent nonconductive Kevlar^®^ samples from charging and to enhance image quality. A modular program for SPM (scanning probe microscopy) data visualization and analysis, Gwyddion (CEITEC, Brno University of Technology, Brno, Czech Republic), was used for AFM image processing and to obtain quantitative data.

### 2.7. Statistical Analysis

Data describing all the options were expressed as mean values ± confidence intervals. Fisher’s tests, t-tests and one-way analyses of variance were performed (using Microsoft Office Analysis Tool Pak file ANALYS32.XLL for statistical and engineering analysis) to compare the modified fabric variants with the corresponding unmodified one; F_calculated_ > F_crit_ (1; 38) and *p* < 0.05 were considered significant. The effects of laser power and scanning speed on yarn pull-out force are described with the regression model approach using experimentally obtained average JRF values following a randomized experimental design 2^2^ with two replicates. The regression models were used to generate response surfaces in terms of the natural factor levels within the laser power and scanning speed intervals under investigation. Corresponding response surfaces and contour plots were used to identify and compare areas with the highest JRF values obtained as a result of laser pre-treatment of KM2+440 fabric and the subsequent deposition of two graphene layers.

## 3. Results

Kevlar^®^ KM2+440 and KM2600 fabric samples were prepared by performing pre-treatment using laser parameters according to the variants specified in Table 2. After laser pre-treatment, the fabric samples were subjected to the technology of graphene deposition, creating one- (designation 1k) or two-layer (designation 2k) coatings following the experimental design specified in Table 3. By tracking the changes in JRF (P_max_) of the samples treated sequentially according to the KM2+440 and KM2600 fabric sample processing plans (Table 3) and relating the measurement results obtained at each next step to the value of the corresponding unmodified sample, synergistic effects were evaluated, which manifest differently depending on the set of laser treatment parameters used and the type of Kevlar^®^ fabric. The graphene deposition technology is the same for all variants under discussion.

### 3.1. Results of Kevlar^®^ KM2+440 Fabric JRF Tests

The results of the processed Kevlar^®^ KM2+440 JRF tests obtained using quasi-static yarn pull-out test methodology are shown in Table 4. The data in the first row of Table 4 refer to the unmodified reference sample. In the last column, increased percentages are shown to evaluate the relative contribution of each treatment.

The JRF increase is calculated according to the formula:

[JRF(Vi, j) − JRF(0)]/JRF(0)(1)

where the JRF of unprocessed fabric is JRF_1_(0) for KM2+440 fabric and JRF_2_(0) for KM2600,

JRF of processed fabric JRF(Vi,j), Vi,j variant designation, i = 0…5,

j = 1…6 − accordingly sequential processing: laz, laz1k, laz_2k, 1k, 2k, 3K.

**Figure 7:**
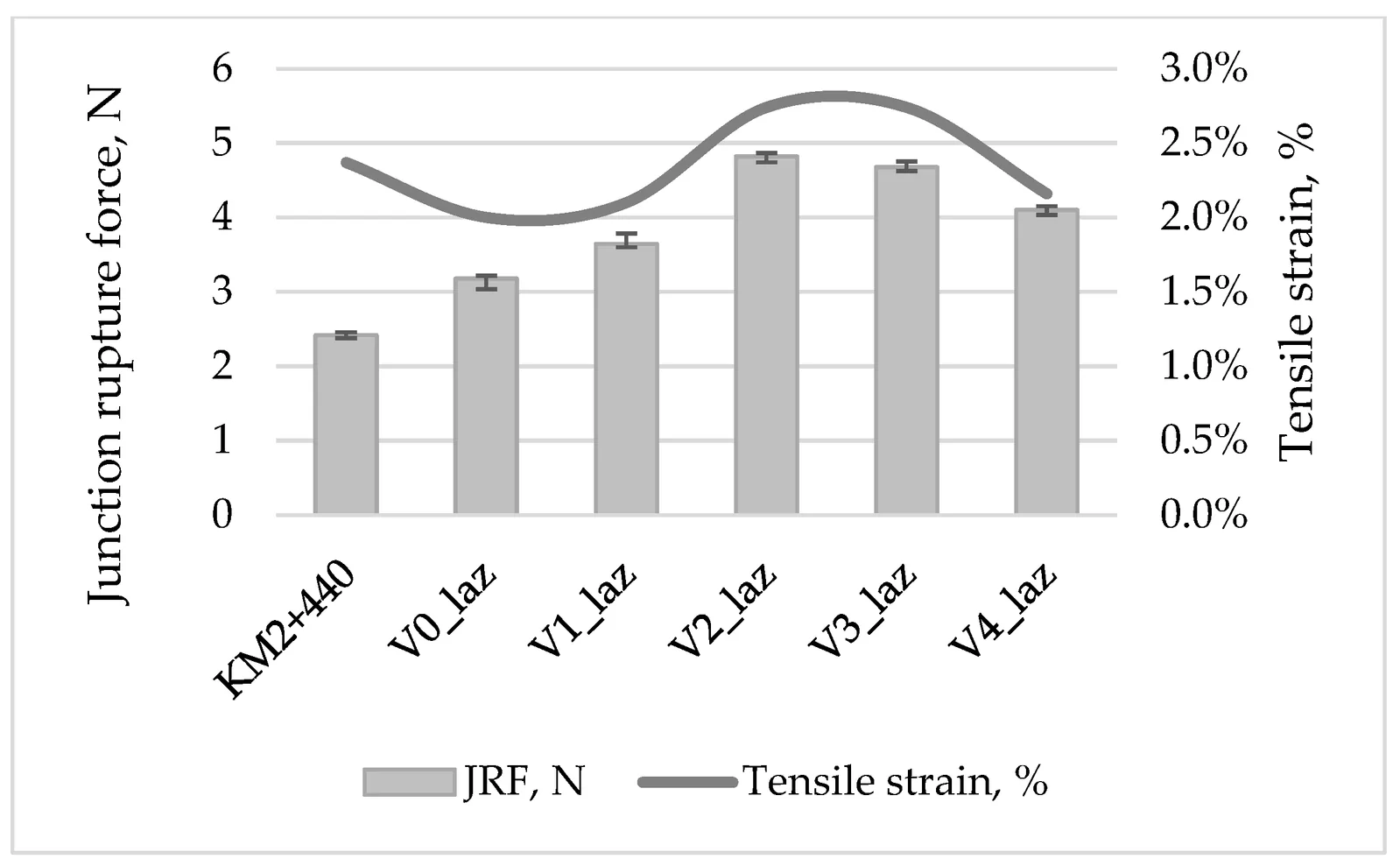
Junction rupture force and tensile strain changes after treatment with a (CW) CO_2_ laser depending on beam power and scanning speed at a raster step of 80 µm.

Table 4 shows that the laser pre-treatment of the KM2+440 fabric surface within the applicable power and scanning speed limits increases the average JRF values by 32% to 99% compared to the average JRF values of equivalently pre-cleaned untreated samples. Figure 7 shows that the set of laser parameters corresponding to variants V2KM2+440_laz and V3KM2+440_laz cause the largest improvement in average JRF values, to 99% and 93%, respectively, as shown in the last column of Table 4. Tensile strain variations within 2% do not indicate that the changes depend on the examined laser parameters and rather serve as an indicator for monitoring the yarn pull-out process.

The JRF pull-out test mean values confirm the conclusions of previous experiments measuring fabric surface roughness—changes in surface morphology are greater when combining lower power values within the observed range (Table 2) with higher scanning speeds, or higher power with lower scanning speeds. The latter combination, however, is risky and only works with very small deviations from the extreme points of the parameter change range. The authors Nofal et al. [47] reached similar conclusions when studying polyester and cotton textiles at the LST macro level, which is not surprising since a common property of para-aramid fibres, including Kevlar^®^, as well as polyester and cotton fibres, is that they do not melt when heated but rather carbonize when the power threshold is exceeded.

**Figure 8:**
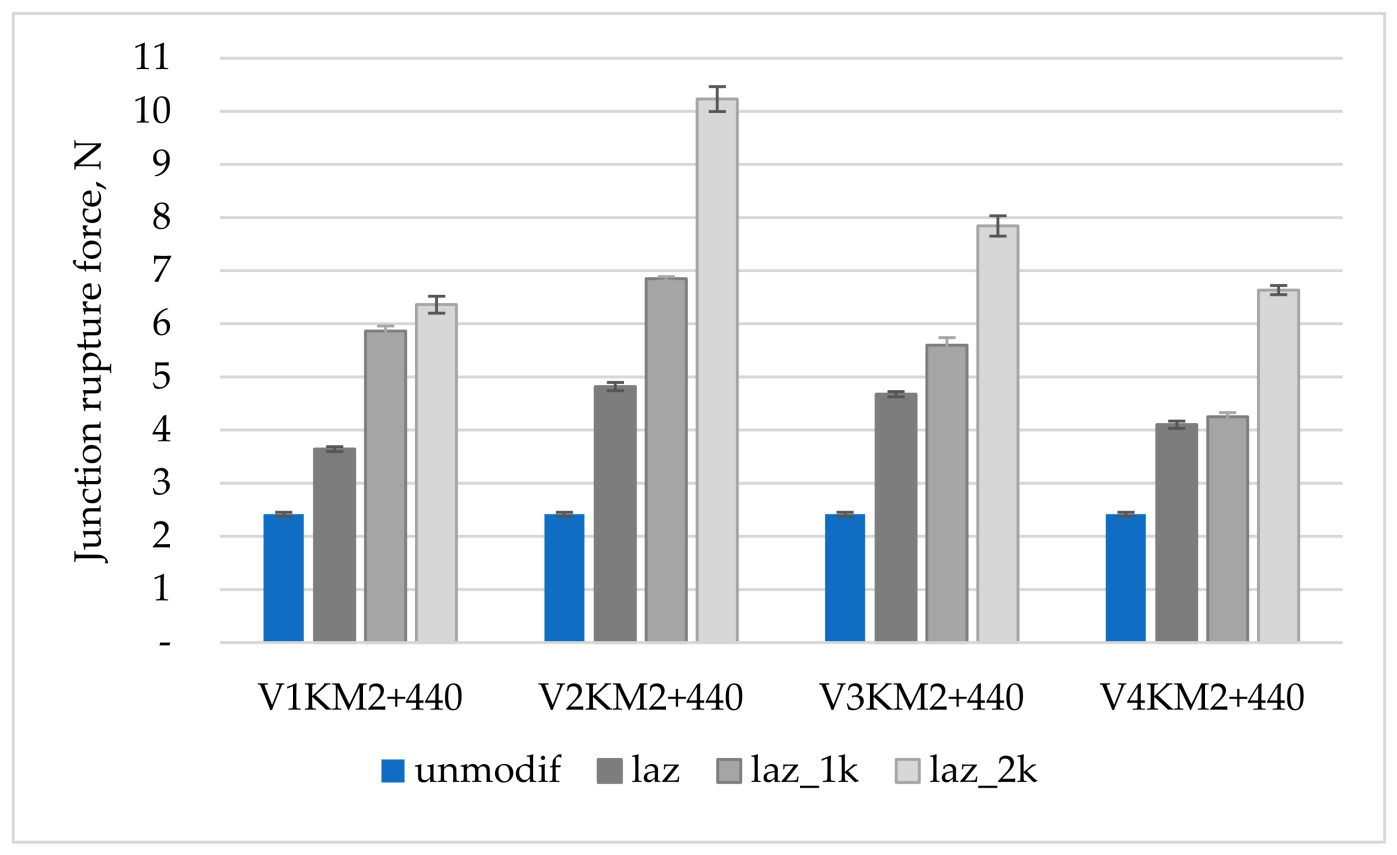
Effects of laser pre-treatment on the JRF of graphene-modified KM2+440 fabric samples.

The particularly pronounced effect of laser pre-treatment is visible in the average JRF value of the V2KM2+440 and V3KN2+440 samples modified with the following graphene coatings (Figure 8), especially when two graphene layers were deposited, V2KM2+440_laz_2k and V3KN2+440_laz_2k. The results indicate that the increase in the average JRF value of the graphene-modified samples follows the same upward trend as the laser pre-treatment JRF values.

Equation (2), obtained following the standard data processing procedure of the 2^k^ Experimental Design, describes the experimental results adequately without residues, as the values predicted by the regression (2) match the experimental JRF_laz_ values obtained by laser power and scanning speed combinations determined by the experimental plan 2^2^. The equation indicates that the power and scanning speed interaction effect dominates the linear effects, as shown by the coefficient ratios in Equation (2).

JRF_laz_ = 4.31 − 0.15x_1_ − 0.08x_2_ − 0.44x_1_x_2_ (2)

**Figure 9:**
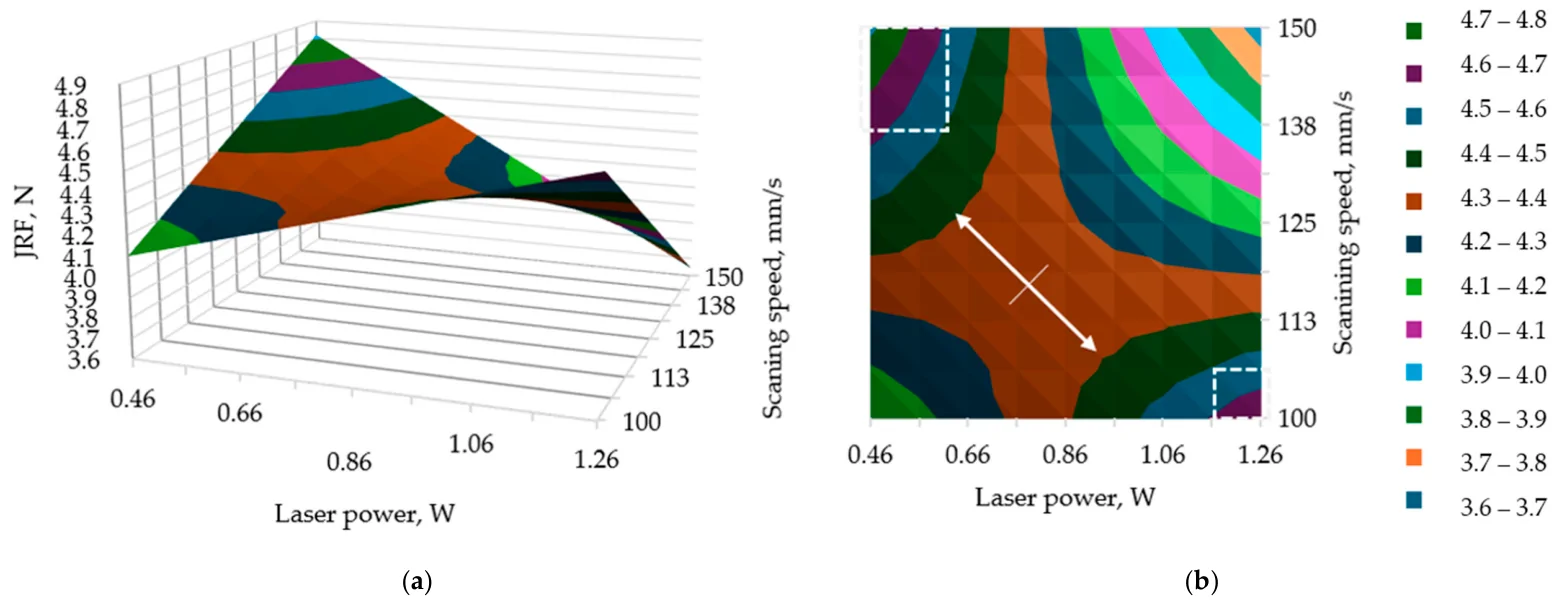
Influence of laser pre-treatment parameters on the KM2+440 fabric JRF; (**a**) response surface; (**b**) contour plots.

Since there is a “mini-max” point in the centre of the response surface, the highest JRF value within the experiment range of 4.5–4.8 N can be achieved in the area of 0.46–0.66 W combined with SS 138–150 mm/s (the area marked in the upper left corner), as well as by combining P in the range of 1.16–1.26 W with SS 100–107 mm/s (the area marked in the bottom right corner) (Figure 9a,b). This trend correlates with the response surface contour plot of surface roughness increase in relation to the raw samples obtained in the authors’ previous studies, which were obtained under the same laser processing parameters [23].

Equation (3), which describes the yarn KM2+440 fabric with laser pre-treatment and the subsequent graphene two-layer coating KM2+440_laz_2k JRF changes under the influence of laser pre-treatment, indicates that the power and scanning speed interaction effect still dominates.

Y_laz_2k_ = 7.77 − 0.67x_1_ + 0.53x_2_ − 1.27x_1_x_2_ (3)

**Figure 10:**
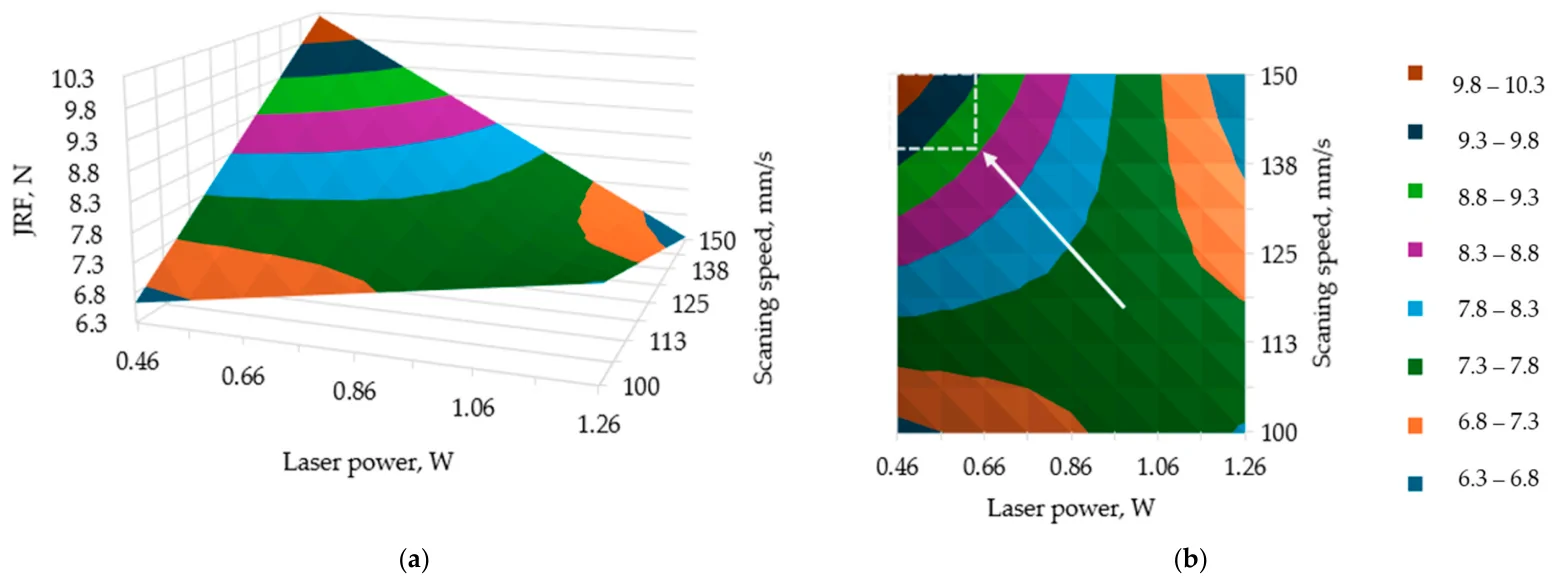
Laser pretreated graphene-coated KM2+440_laz_2k fabric JRF; (**a**) response surface; (**b**) corresponding contour plots.

Corresponding response surface and contour plots show the highest JRF increase for graphene-coated KM2+ fibre fabric within the experimental range of 8.8–10.3 N, which can be achieved in the area of 0.46–0.66 W combined with SS 138–150 mm/s (the area marked in the upper left corner), and the same trend persists only after laser pre-treatment (Figure 9). But the highest increase range of JRF, unlike pre-treatment, is not provided in the range of 1.16–1.26 W with SS 100–107 mm/s (the area in the upper right corner) (Figure 10a,b). This suggests that the applied combinations of P and SS produce different effects on the fibre surface—less intense laser exposure creates bumps, while more intense exposure results mostly in deep cavities, which have less impact on the graphene sheets’ adhesion.

It is possible that the obtained high yarn pull-out resistance could be strongly influenced by the properties of the KM2+440 fabric structure. It is a relatively light, tightly woven plain weave ballistic-grade fabric (14 threads per cm in the warp and weft directions, thickness of 0.17 mm, areal density of 125 g/cm^2^) with a relatively smooth surface and good laser beam access to aligned fibres on yarn overlaps.

### 3.2. Experimental Results of Processed Kevlar^®^ KM2600 JRF Tests

For comparison, the effects of laser pre-treatment on KM2600 fabric were examined (600 denier threads, plain weave, 11.2 threads per cm in the warp and weft directions, fabric thickness of 0.23 mm, areal density of 146 g/cm^2^) applying the V2 and V3 variants of laser treatment options (Table 2), which were more effective for the previously analysed KM2+440 fabric.

The results of the yarn pull-out tests of the processed Kevlar^®^ KM2600 fabric samples are shown in Table 5. The data in the first row of Table 5 refer to the unmodified reference sample KM2600. The percentage increases calculated by Equation (1) in the last column of Table 5 show how much each fabric treatment contributes to the JRF increase.

**Figure 11:**
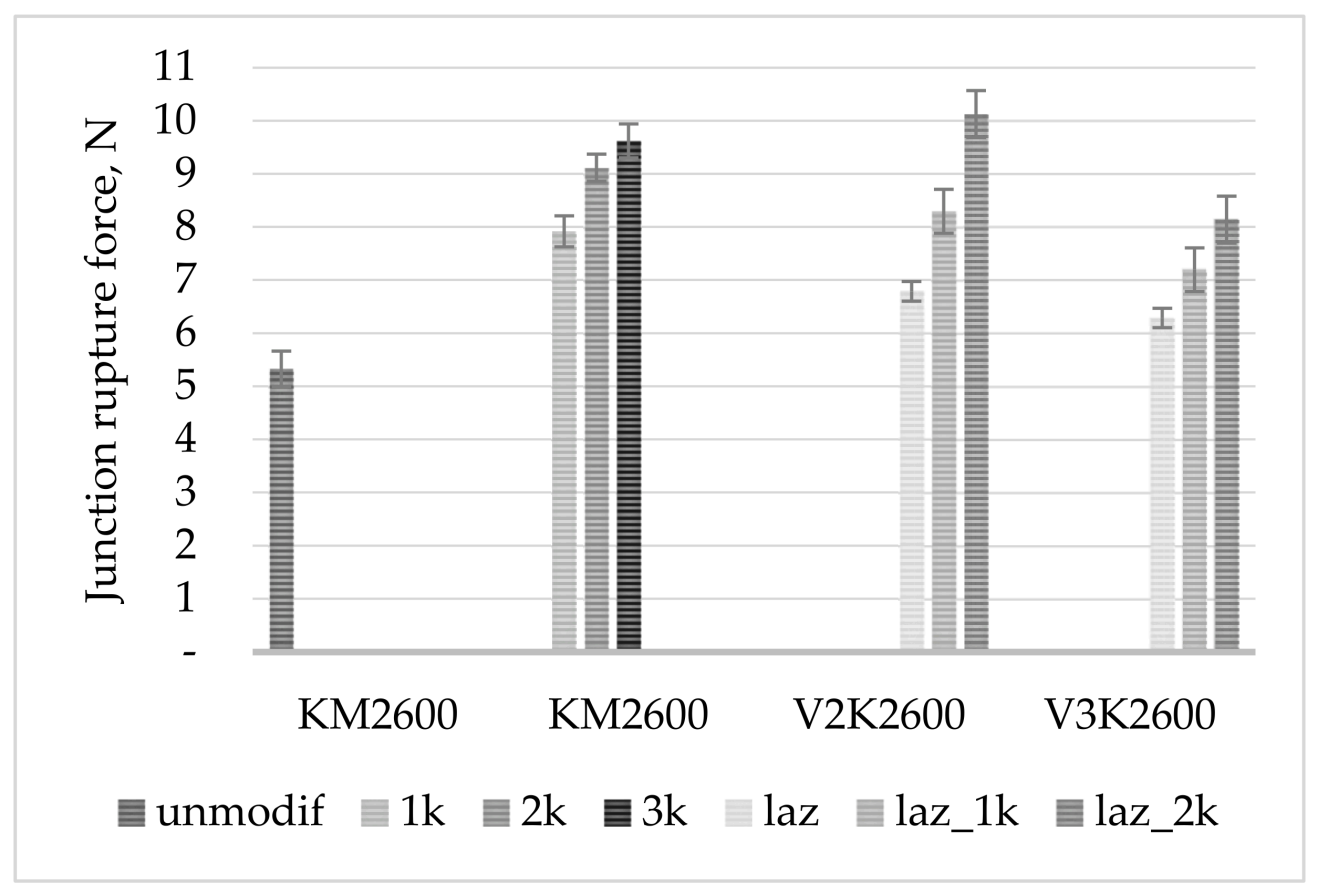
Effects of laser pre-treatment on graphene-modified KM2600 fabric sample JRF values.

**Figure 12:**
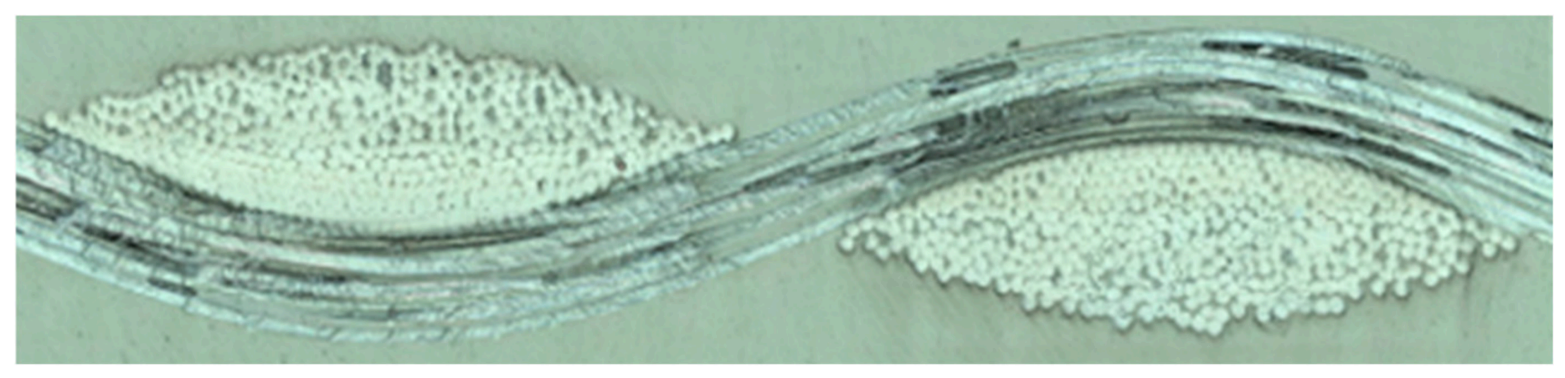
Micrograph of the KM2600 warp yarn cross-section containing 400 filaments with an approximate diameter of 12 µm [49].

Comparing the percentage changes in JRF shown in Table 5 and the changes in average JRF values shown in the graph (Figure 11) depending on the applied modifications, it is evident that the effect of laser pre-treatment is significantly weaker compared to KM2+ fibre fabric. As a result of laser treatment, the V2KM2600_laz variant JRF increased by 27% compared to the unmodified fabric, while the differences in the V3KM2600_laz variant JRF (18%) were almost statistically insignificant, and any further JRF increase in V3 was only provided by graphene coatings. Meanwhile, for the laser-modified V2KM2600_laz with a single graphene coating layer, JRF increases by 56%, and adding a second graphene layer boosts it to 90%, even exceeding the KM2600_3k variant JRF without laser pre-treatment, which increases by 81%. These obvious differences in JRF growth between fabric types under discussion can be partly explained by the different fabric structure parameters, especially their thread densities of 14 and 11 threads per cm, respectively, and fabric densities of 28 and 22 treads/cm^2^, correspondingly. Relative fabric density represents how full the fabric is compared to the maximum packing density allowed by its weave, providing a more accurate basis for comparison than absolute fabric density. Analysis revealed that both the type of weave and relative fabric density have a statistically significant effect on surface roughness at the macro level [48]. The relative density of KM2600 fabric is lower compared to KM2+440 fabric because it has fewer threads per cm and a much larger number of filaments in the cross-section of the KM2600 yarns (Figure 12), which provides a greater opportunity for the thread-forming filaments to rearrange during the fabric formation process.

As seen in the micrograph, the filaments are randomly packed together with no apparent order. Filaments in the cross-section that are situated closer to the orthogonal weft yarn are packed together tightly in what appears to be a hexagonal close packing configuration, while the filaments closer to the free surface of the fabric are loosely packed together [49]; due to the curvature of the yarn surface, they are at different distances relative to the laser beam’s focal plane. A comparative analysis shows that differences in fabric architecture affect the effectiveness of the applied modifications if the set of processing parameters does not change, but the trends in response to a certain combination of laser parameters remained in both the Kevlar^®^ fibre KM2 and KM2+ fabric variants being compared.

The purpose of the applied modification is not to create changes on the macro level of the fabric surface, which occur, for example, in engraving or welding processes. During the experiment, the sets of laser processing parameters applied can change the very smooth surface of Kevlar^®^ fibres, creating nano- and/or microstructures on them to ensure better adhesion with coatings or matrices while maintaining the mechanical and other application-relevant properties of fibres. However, to achieve this, it is necessary to coordinate the laser processing parameters very precisely when working with fibre materials whose structural stability is very sensitive to the laser beam’s effect.

## 4. Discussion

### 4.1. Self-Organization of Laser-Irradiated Nano- and Microstructures

The experimental data show that, within the tested range, using the lowest laser power with the fastest scanning speed gives the highest JRF values, both after laser pre-treatment and after adding one or two graphene layers (Figure 9, Figure 10 and Figure 11). This seems a bit counterintuitive at first, but it makes sense once you look at what kind of surface changes actually give the expected results. Currently, several theories are being considered that provide an opportunity to describe surface structures that arise when a laser beam acts on a material’s surface. When studying the effect of laser processing on polymer surfaces, theories of light-induced material reorganization are considered more suitable. Material reorganization theories focus on reshaping laser-irradiated material into a topography characterized by a periodic combination of grooves and ridges. The mechanisms underlying this theory, which are currently under investigation, include thermodynamic phase transitions, hydrodynamic effects of the transiently activated surface, unstable phases of the material during irradiation, accumulation of defects, diffusion, and surface erosion. Molecular Dynamics (MD) modelling applied to these effects predicts the kinetic trajectory of an ensemble of atoms as the laser interacts with the solid. This is achieved by numerically solving Newton’s equations of motion spatially and temporally at the atomic scale [50].

Solutions rely on local surface gradients, which require the surface to remain in an unstable, metastable state for a sufficient time interval to induce material reorganization. Usually, three laser irradiation regimes are considered to differ by the laser fluences needed for nano- and micro-structuring (NMS) of surfaces. In the regime of a relatively low subthreshold fluence, NMS formation results from multi-pulse irradiation in the absence of the melting of a solid [51]. The most salient feature for all irradiation types and regimes is the universal behaviour of the lateral size of the structure, which can be interpreted as a linear dependence of the lateral size of the nano- and microstructures on the thickness of the defect-enriched layer formed due to energy deposition. This experimental dependence is revealed for all irradiation types and regimes. The irradiation results in the formation of a nanometre-thick subsurface defect-enriched stressed layer **h**. The nature of this nanolayer, its thickness and the stress in it, and the defect types depend on the material properties and the irradiation regime and type, which must be determined in each case.

Defects in layer **h** have enhanced the lateral mobility due to the nonequilibrium conditions for defect generation (charging of point defects, heating, a relatively high level of plasma excitation, ion-stimulated and stress-enhanced diffusion, etc.). The thickness **h** of the defect-enriched nanolayer serves as the first scaling parameter that determines the NMS lateral dimensions [51].

**Figure 13:**
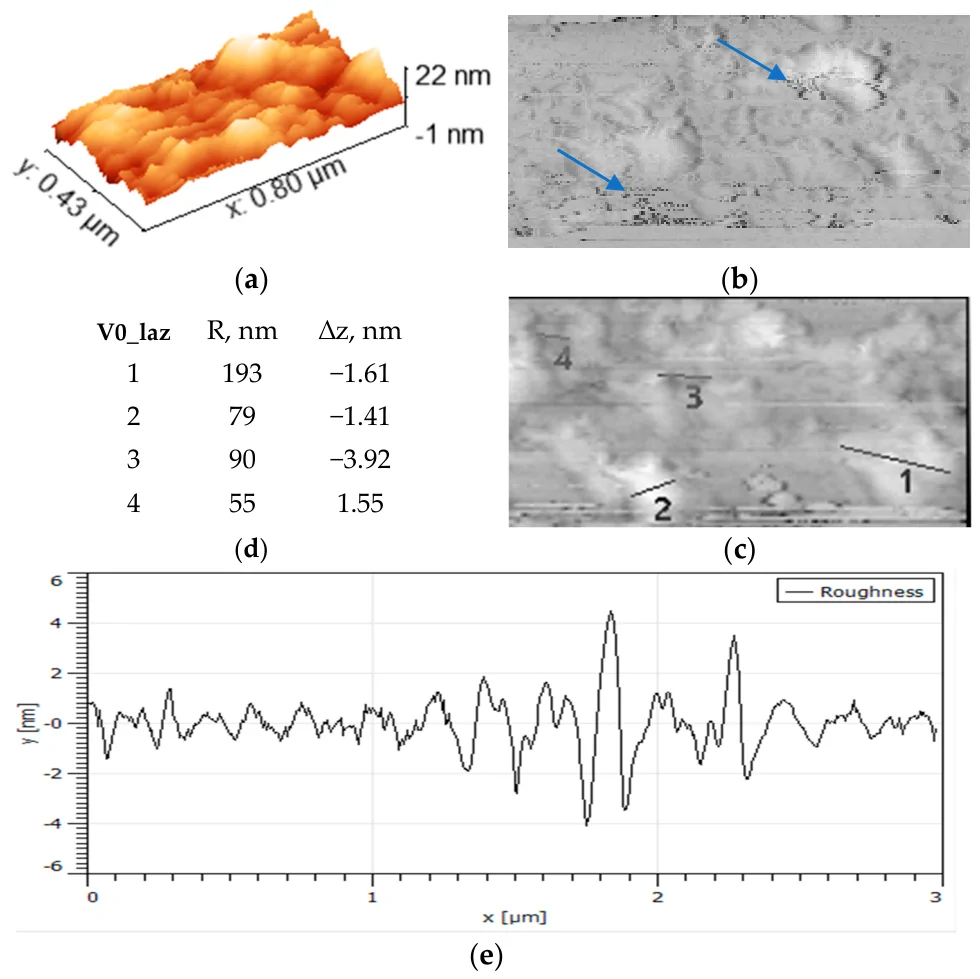
AFM micrographs of Kevlar^®^ KM2+440 fabric variant V0_laz and effects of surface laser processing. (**a**) 3D AFM image at the nanoscale; (**b**) point-size defects in layer **h** formed individually or in smaller or larger groups around them (blue arrows); (**c**,**d**) numbers indicate from which flakes the parameters particle lateral size R and elevation ∆z are measured; (**e**) corresponding fibre surface nanoscale roughness graph.

**Figure 14:**
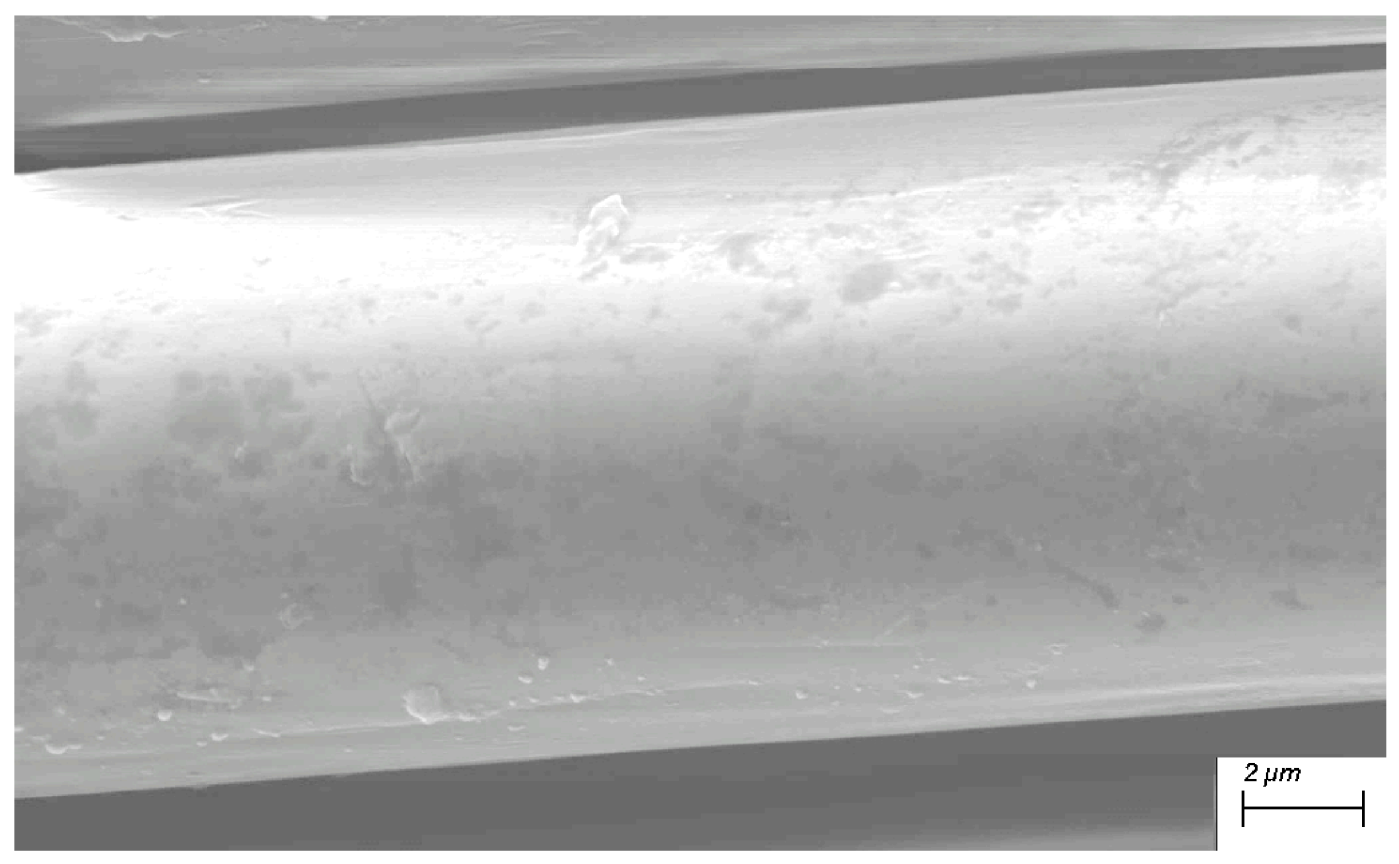
SEM micrograph of V0KM2+440_laz. Mag., ×5000.

The peak-to-valley height of laser-processed KM2+440 fabric variant V0_laz does not exceed 23 nm (Figure 13a). This means that the surface roughness changes obtained as a result of the laser treatment are located in the fibre skin and do not affect its core structure, as the KM2 fibre skin thickness is estimated to be in the range of 300–350 nm [52,53]. Point defects in layer **h** form individually, but most often in smaller or larger groups, as shown by the blue arrows (Figure 13b), and around them, nanoscale bumps and cavities are formed (Figure 13a,b). The lateral size R of defects measured from AFM micrographs range from a few tens to a few hundred nanometres (Figure 13c,d) (Figure 14). The average thickness of the defect-enriched nanolayer KM2+ fibres is estimated at **h**~8 nm (Figure 13d,e). This means that it is the upper layer of the KM2+ fibre skin, whose thickness is less than a micrometre.

The average surface roughness Sq = 5.8 nm and the corresponding surface area increase of 4.8% after laser treatment have ensured a 32% increase in the average JRF of the V0KM2+440_laz variant (Table 4). The surface is mainly covered with shallow cavities whose lateral size varies from a few tens of nm to a few hundred (Figure 14).

**Figure 15:**
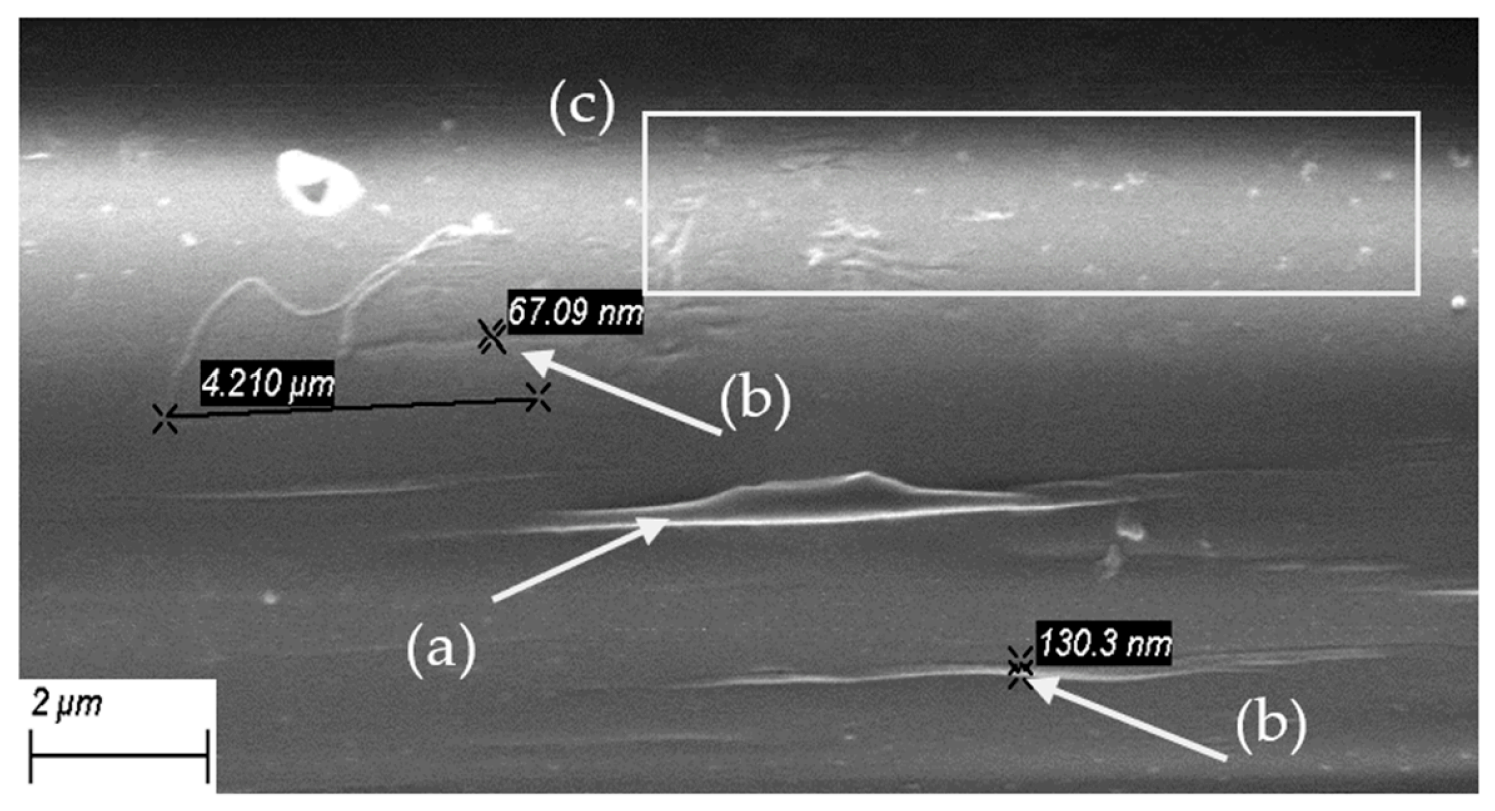
SEM image of the laser-pretreated V2KM2+440_laz fibre surface. (**a**,**b**) Grooves of different types running along the fibre’s axis; (**c**) single and grouped nanoscale bumps. Mag., ×6000.

Grooves ranging from 4 to several hundred micrometres in length are located on the fibre surface of the V2KM2+440_laz processed fibre along the axis direction (Figure 15a,b), with their widths varying from a few tens to a hundred nanometres, accompanied by nanoscale bumps (Figure 15c), providing a surface roughness of Sq = 6.7 nm and an increase in surface area by 14.7%.

With these light fibre skin surface modifications, the JRF increase to 99% (Table 4) is very significant, possibly because micro-sized grooves are combined with nano-sized bumps.

**Figure 16:**
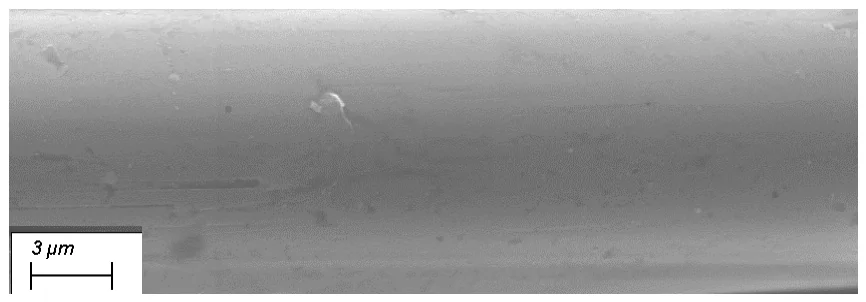
SEM image of V0KM2600_laz. Mag., × 300.

The motion of defects from the surface nanolayer along the normal to the surface (diffusion or strain-induced drift) leads to a delayed formation of the second defect-enriched layer with thickness **H > h**. The thickness **H** of this expanded defect-enriched layer represents the second scaling parameter. This parameter determines the lateral large-scale modulation of the surface relief that emerges after a relatively long irradiation and is imposed on the nanometre-scale relief. In the case of the generation of micrometre-scale surface structures, parameter **H** determines the period of the structures [50,51]. Initially, the mobile point defects are uniformly distributed along the surface. A fluctuating local increase in the surface defect concentration gives rise to the corrugation of the surface relief and the corresponding surface strain. The defects interact with this long-range surface strain field owing to the deformation potential. The strain leads to a lateral defect flux that is proportional to the defect concentration and is oppositely directed with respect to the diffusion flux. At a relatively low defect concentration, the diffusion flux exceeds the strain-induced flux, the fluctuations in the spatial defect distribution decay, and the surface remains almost flat (Figure 16).

**Figure 17:**
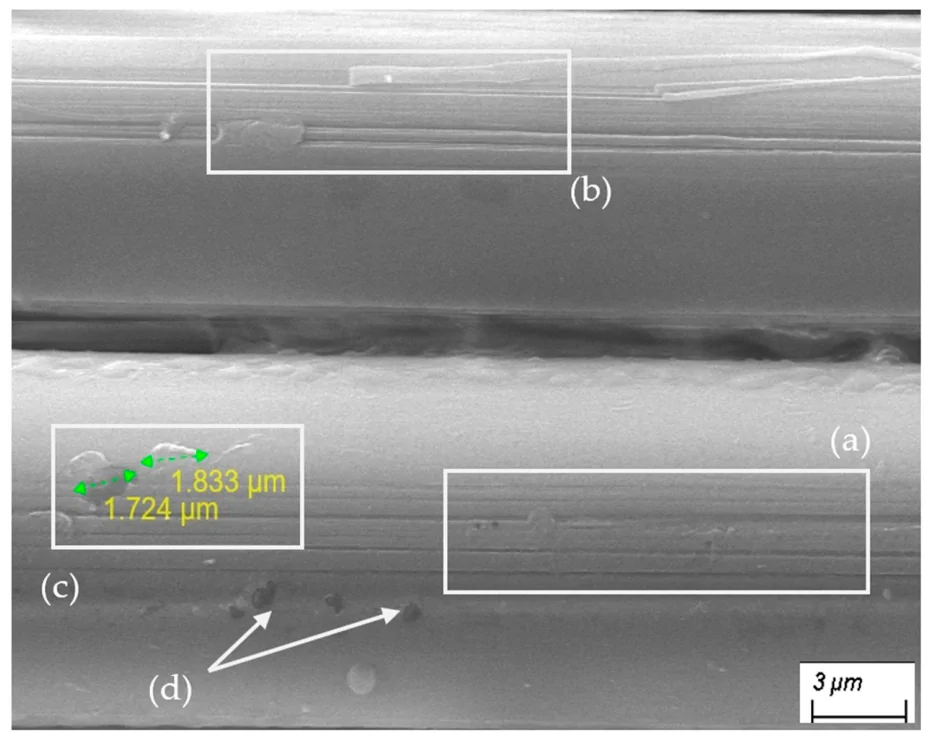
SEM image of the laser-processed V2KM2600_laz fibre surface. (**a**,**b**) A spatially periodic, corrugated surface structure oriented in the fibre axis direction; (**c**) the periodically corrugated surface combined with cavities of lateral size 1.7–1.8 µm; (**d**) surface defects in the form of regular wells. Mag., ×3000.

The second stage of the surface structuring starts when the concentration of mobile defects exceeds a certain critical level. At this critical point, the lateral strain-induced defect flux starts to exceed the lateral diffusion flux. From the viewpoint of the universal defect–deformation (DD) mechanism, the creation of these structures leads to an instability in which defects are autolocalized in periodic self-consistent strain wells (Figure 17c,d). Thus, the homogeneous lateral distribution of the defects is transformed into a spatially periodic (along the surface) distribution. This transformation is accompanied by periodic corrugation of the surface; with the defect piling up at the extrema of the surface relief, a ridged surface structure is formed (Figure 17a,b). Grooves typically occur in areas of the surface where a significant amount of material has been removed. The preferential orientation of grooves is generally parallel to the polarization direction of laser radiation, although in rare cases, grooves can be oriented orthogonal to the polarization [54].

By modifying the V2 variant Kevlar^®^ KM2600 fibre fabric with laser pre-treatment and applying two layers of graphene coating, its JRF increased compared to the as-received fabric for surface fibres by 27% after laser treatment, 56% after applying the first layer of graphene, and 90% after applying the second layer (Table 5, Figure 11).

**Figure 18:**
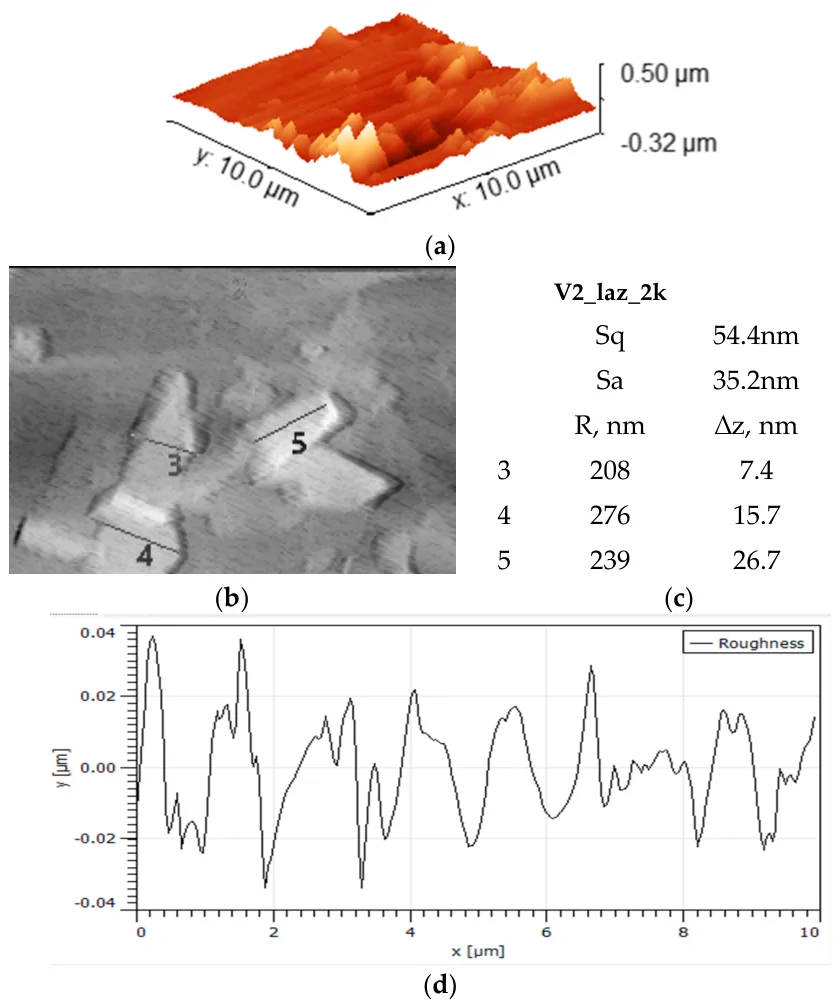
AFM micrographs of Kevlar^®^ KM2600 fibres of fabric variant V2_laz_2k and effects of surface laser processing. (**a**) 3D AFM micrographs over a scan area of 10 µm × 10 µm; (**b**) deposited graphene flakes on the laser pretreated fibre surface; numbers indicate from which flakes parameters R and ∆z were measured; (**c**) surface RMS (Sq) and RA (Sa) roughness, and particle lateral size R and elevation ∆z measurements; (**d**) fibre surface nanoscale roughness graph.

The average surface roughness Sq = 54 nm of the sample modified with a double graphene layer coating is significantly increased by multilayer graphene sheets (Figure 18a,b), with lateral sizes in the range of 200 to 300 nm and highest peaks less than 40 nm (Figure 18c,d).

**Figure 19:**
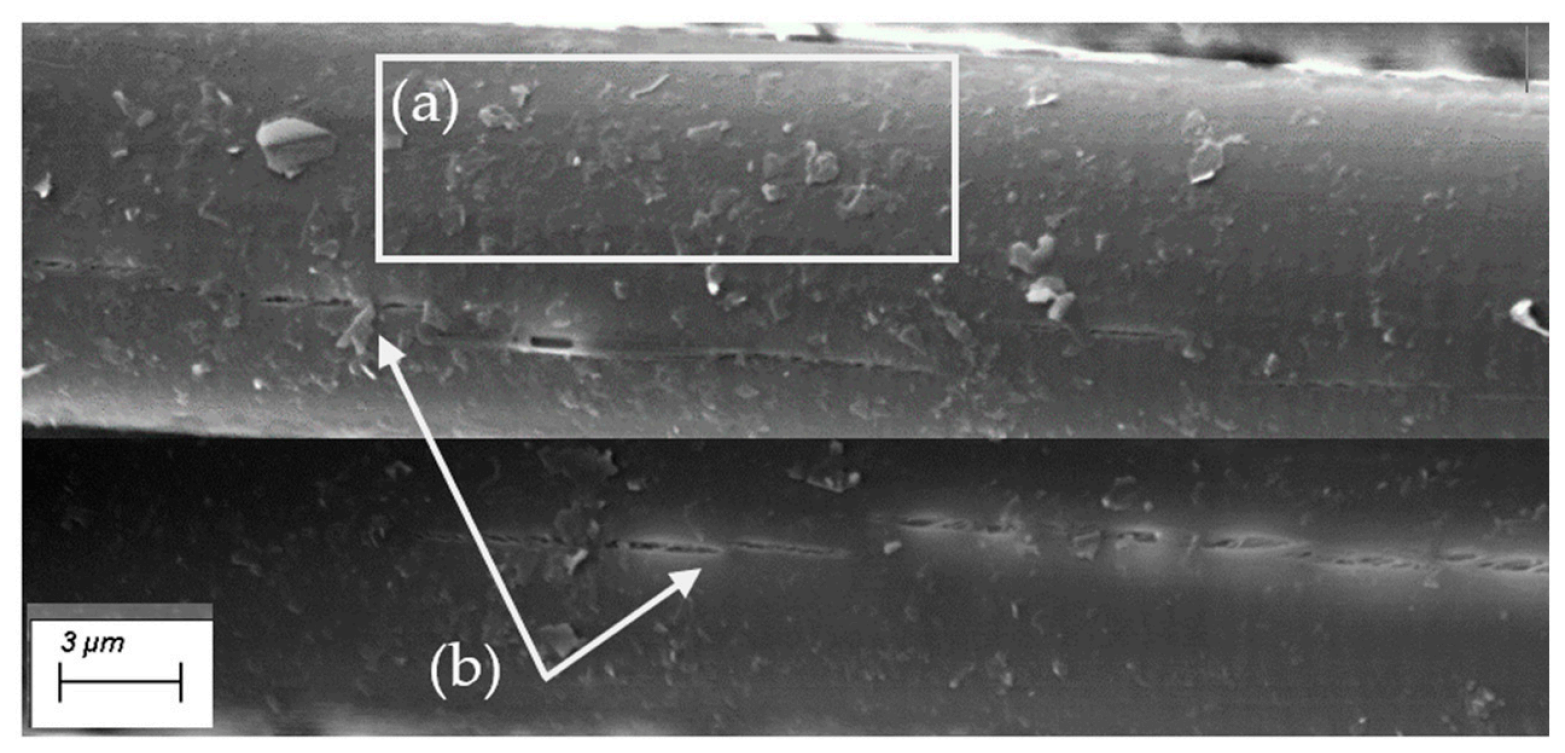
SM image of laser-pretreated V3KM2600_1k modified with one layer of graphene coating. (**a**) The deposited graphene flakes anchored on nanoscale bumps created by the laser pre-treatment; (**b**) the deposited graphene flakes clustered around and cross the grooves created by the laser on the fibre surfaces. Mag., × 3000.

Figure 19 provides a brief insight into the interaction between laser processing and the single-layer surface modification using graphene. Graphene flakes of different lateral sizes are randomly distributed on the unmodified and laser-beam-modified surface areas with or without covering the laser-irradiated structures. This suggests that both effects are cumulative, which is indirectly confirmed by the increase in V3KM2600_laz JRF from 18% after applying the first layer of graphene to 35% (Table 5). Thus, shallow cavities with a lateral size in the range of a few tens to a few hundred nanometres and grooves along the fibre axis, as well as negligible-sized nano-protrusions, dominate when irradiated with a low-power laser beam. Meanwhile, the previous study showed that the results of combining high power with low speed are dominated by deeper fibre surface structures [23], which increases the measured roughness values but does not give the expected JRF increase from laser pre-treatment and graphene layer deposition, suggesting that the laser-induced fibre surface topography may contribute significantly.

Using the optimal set of laser parameters, the obtained V2KM2+440 and V2KM2600 variant JRF increases (Table 4 and Table 5) clearly confirm the benefits of treating Kevlar^®^ fabrics with a low-power CW CO_2_ laser beam. At the same time, the allowable low power is an obstacle when organizing industrial processing.

**Figure 20:**
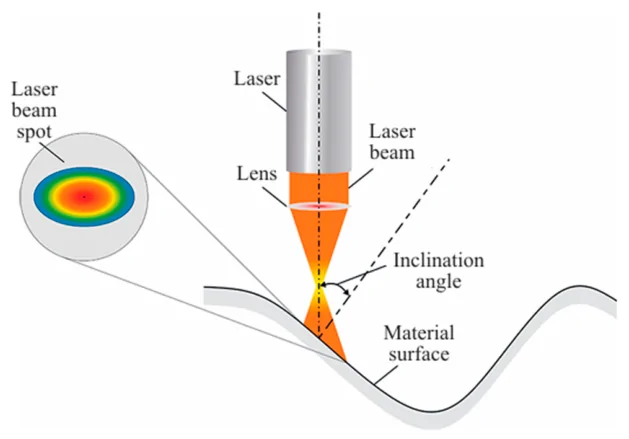
Uneven material surface and inclined beam defocus laser beam spot with an eclipse shape and low laser energy intensity [55].

An unstable focus or beam waist position on an uneven surface (Figure 20) results in fluctuating laser energy density in the irradiated area; subsequently, areas with varying intensities of the produced topographic effects on the fibre surface are formed (Figure 19), which can compromise the precision and quality of the processed surface. However, maintaining the stability of the focus beam position in laser processing is not easy in micromachining and surface processing [55]. Moreover, the CO_2_ laser power suitable for experiments, i.e., 0.46–1.26 W, is low for fabric pre-treatment in industrial production.

### 4.2. Effects of Laser Beam Defocus

Defocused laser beams have been used in laser cutting and welding for several decades [56,57,58]. Setting the focal plane below the surface can lead to deeper welding beads, thus increasing productivity in keyhole welding. Two types of defocus are observed in laser processing: positive and negative defocus. Positive defocus occurs when the laser beam focuses above the material surface, aligning with the position of the focus beam. Conversely, negative defocus occurs when the focus is below the work piece or material surface, or the focus beam is positioned beneath it.

The authors of study [59] aimed to investigate laser beam focus shift (defocus) to increase the laser spot size in selective laser melting (SLM) of 316L stainless steel powder. They found that within a single laser setup, implementing defocus can lead to a substantial increase in potential process productivity. Comparing positive and negative defocus distances, negative defocus leads to a deeper melt pool due to its convergent nature. Positive defocus is less energy-efficient but also less sensitive to heat input variations, resulting in a more stable SLM process [59]. Studies of laser powder bed fusion (LPBF) indicate that modulation of the defocus distance to adjust the beam spot size is critically important and technically feasible for achieving high-quality LPBF components [60].

Based on laser beam focus shift studies in metal technologies and additive manufacturing, some authors hypothesized that the crystalline structure of para-aramids could respond similarly. In the case of positive laser beam defocus values, shallow surface structure changes (conductive node) are formed, whereas if a negative beam defocus is applied, in addition to changes in the skin layer, deeper fibre core structures are affected (keyhole mode) [59]. Regarding fibre polymers, such studies have not been found in literature reviews to date; considering the very uneven surface topography of woven textiles, they can be resource-intensive. However, extensive research is needed to successfully modify the smooth para-aramid fibre surface according to the intended use, including in composite technologies using the “laser marking on the fly” principle, which is already implemented in the CO_2_ roll-to-roll industrial textile texturing system (Figure 1).

### 4.3. Some Effects of Laser Beam Defocus Experimental Application

**Figure 21:**
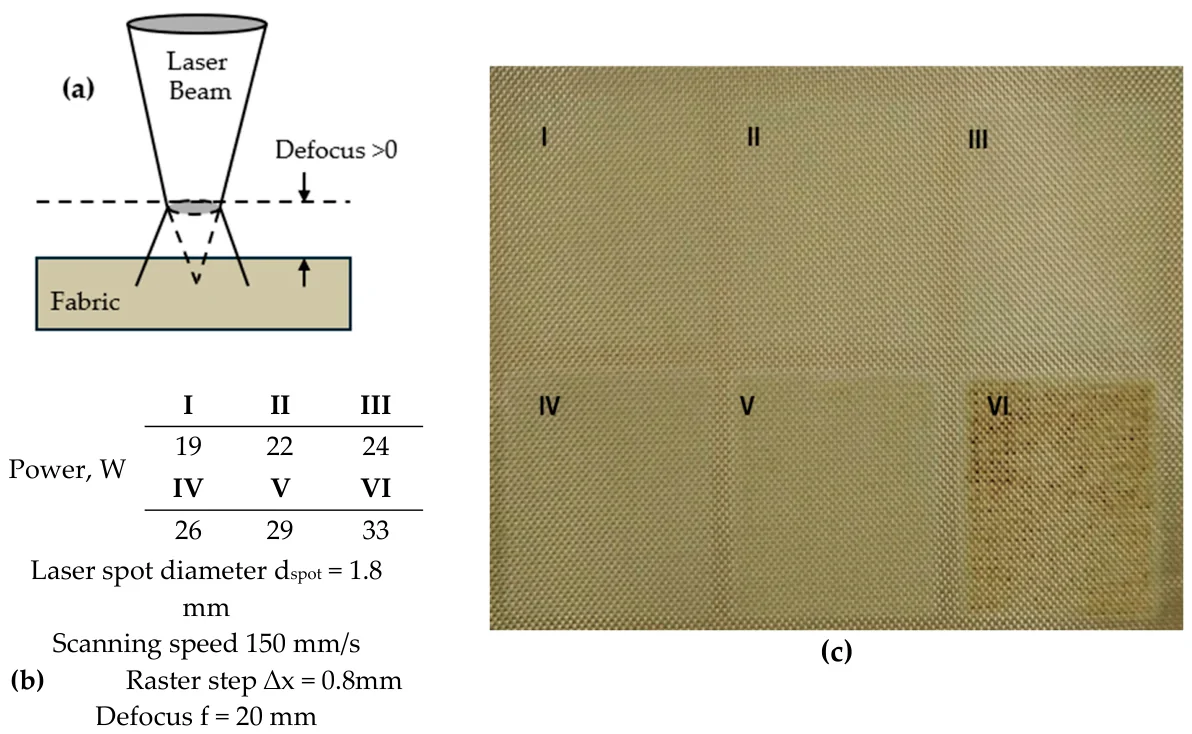
Kevlar^®^ KM2600 fabric samples irradiated by a laser defocused beam in the power range of 19 to 33 W. (**a**) A positive defocused laser spot is intentionally moved away from its focal point to increase the spot size and lower the peak energy density at the surface; (**b**) six suitable laser power options were applied in the range from 10 to 33 W by fixing the defocus 20 mm, scanning speed 150 mm/s, and raster step 0.8 mm; (**c**) images of samples treated with a 1.8 mm diameter defocused laser beam.

Considering that by changing the laser beam diameter it is possible to not only increase the process productivity but also control the roughness of the processed surface by applying positive defocus (Figure 21a) and a higher range of laser power 19–33 W (Figure 21b). Six KM2600 fabric samples of the 4 × 4 cm variants were processed (Figure 21c) to explore the feasibility of processing samples in this power range. The max laser power was 100 W. The beam diameter in focus, d = 100 µm, the focal length, F = 63.5 mm, and the defocus value, f = 20 mm, were kept unchanged (distance from the surface of the specimen up to the focal plane is generally defined as the defocus value). Figure 21a,b shows the experimental setup, where the defocus distance is positive as the focal plane is above the specimen surface. It can also be negative when the focal plane is below the specimen surface to produce keyhole effects [61]. The goal of this preliminary study was to find out how much we can increase laser power by processing experimental Kevlar^®^ fabric with a defocused CO_2_ laser beam; the allowable power limit was determined visually and with a confocal laser scanning microscope.

Progressive degradation of the upper fibre layer was observed in samples of variants IV–VI (corresponding P values in a range from 26 to 33 W); therefore, for further detailed analysis, samples modified within a power range of 19 to 24 W were selected (Figure 21a–c).

**Figure 22:**
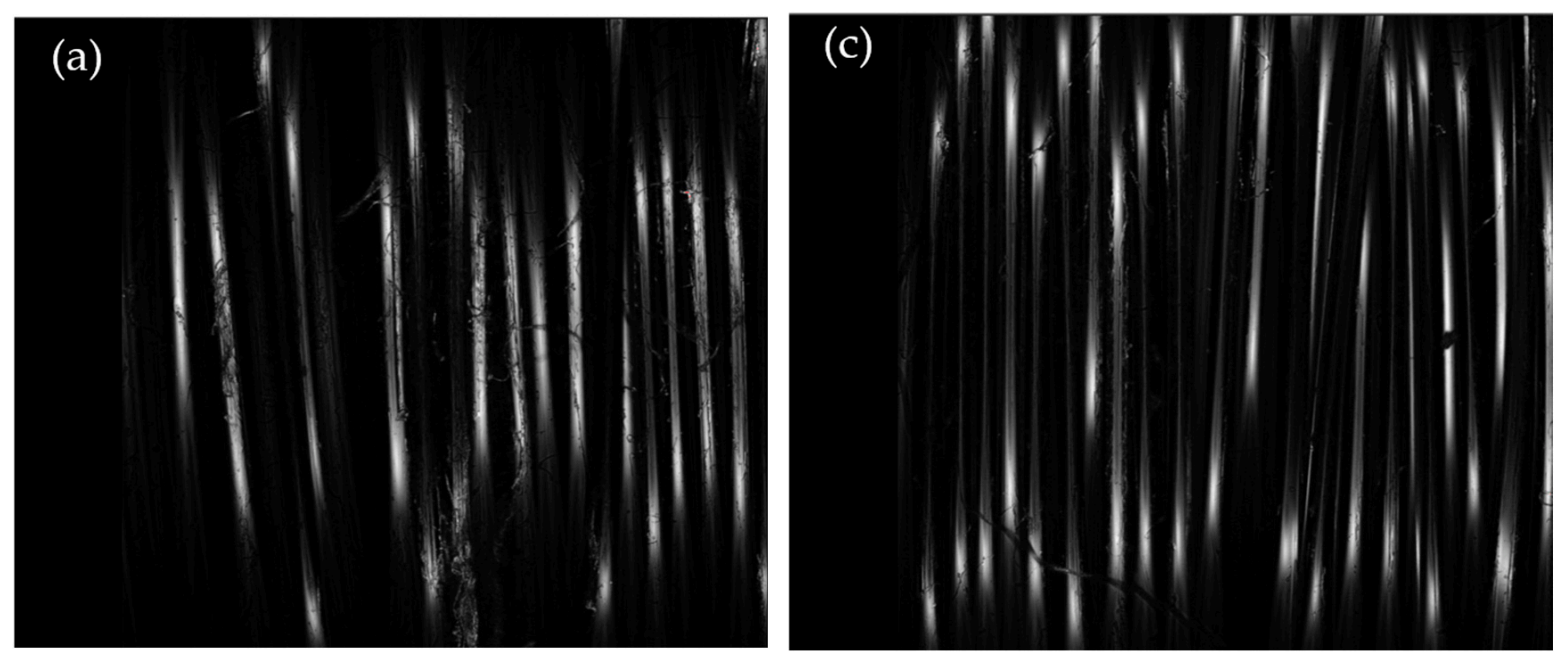

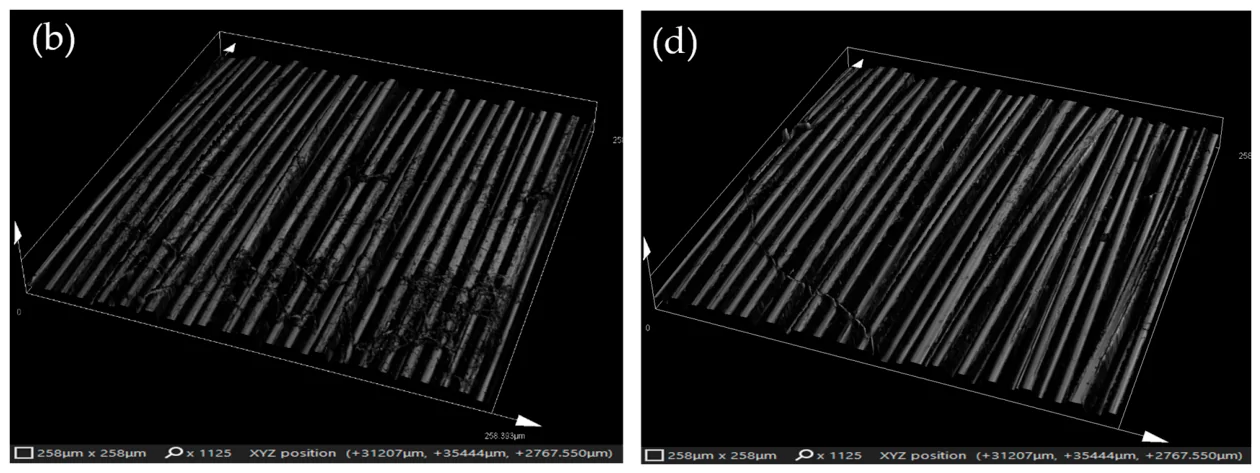
Confocal micrographs of unprocessed (**a**,**b**) and laser-irradiated (**c**,**d**) KM2600 fabric variant III samples; 258 × 258 µm; Mag., ×1125.

The surface of unmodified Kevlar^®^ KM2600 fabric pre-cleaned with acetone is covered with unevenly distributed defects—mostly fibrils and torn skin fragments (Figure 22a,b). Confocal micrographs of fabric variant III laser-irradiated with the parameter set in Figure 21b show clean fibre surfaces without local defects caused by overheating (Figure 22c,d). Laser-irradiated nano/microstructures on fibre surfaces are difficult to see due to insufficient magnification.

To ensure a nano/micro-surface texturing outcome that corresponds to the properties of the textile being processed, the fibre surface structure, and the intended use, a thorough investigation must be carried out to coordinate the set of laser parameters with the defocus values.

## 5. Conclusions

A laser is a flexible tool that allows us to deliver energy to a material at a controlled pace and in a confined area. Depending on the material’s properties and the laser’s settings, different reactions can be achieved in the material, making it possible to develop and optimize processes to constantly change the material’s surface structure so that it fits a desired function. A special aspect in many applications is that material modifications can happen from the atomic to the macroscale, adding complexity to the surface and a new dimension to surface optimization. Laser surface texturing provides a fast, non-contact, and clean alternative for nano- and micro-structuring in ambient conditions. Laser surface treatment has been an important part of many large industrial manufacturing processes, and it continues to evolve and find new uses in emerging areas. As lasers are developed with an ever-wider range of capabilities, laser surface treatment will continue to improve material performance in existing applications and open the door to new materials and new uses that would not be possible without these unique treatment capabilities.

In this work, two types of Kevlar^®^ KM2 fibre fabrics were subjected to CO_2_ laser processing to investigate the influence on yarn pull-out force from fabric structure JRF upon various combinations of laser power P and scanning speed v for a given raster step ∆x = 80 µm and laser spot diameter of 100 µm. Observations and quantitatively determined increases in JRF indicate that small changes in fibre surface roughness induced by laser irradiation of the fabric surface can significantly increase JRF.

Laser processing with a specific set of parameters produces varying intensity effects depending on the characteristics of the Kevlar^®^ fibres and fabric structure, which can be evaluated experimentally. The observations in this study suggest that a relatively smaller number of fibres per yarn and a larger number of yarns per unit in the warp and weft directions of fabrics can significantly increase the JRF of laser-processed fabrics.

Laser pre-treatment combined with a chemical graphene coating provides a synergistic increase in yarn pull-out JRF. Laser nano- and microscale fibre surface modification followed by functionalization with graphene coatings produces cumulative effects, indirectly confirmed by the nearly twofold increase in JRF percentage compared to unprocessed KM2+440 fabric. KM2600 fabric response to yarn pull-out irradiated with the same laser parameter set is more selective and much weaker due to the different yarn and fabric structure parameters.

Comparison of the three-layer graphene-coated Kevlar^®^ KM2600_3k sample JRF without laser pre-treatment with the laser-pretreated, two-layer graphene-coated sample V2KM2600_laz_2k JRF shows that by applying laser pre-treatment, it is possible to achieve equivalent JRF, thus providing the opportunity to reduce the number of very resource-intensive graphene coating layers.

When studying the effect of laser processing on polymer surfaces, theories of light-induced material reorganization are considered more suitable, and this, in relation to laser-irradiated KM2 fibres, is confirmed by preliminary research. The set of laser parameters should be such that the thickness of the defect-enriched nanolayer is much smaller than that of the Kevlar^®^ fibre skin, whose thickness for KM2 fibres is in the range of 300–350 nm, and the resulting surface effects leave the fibre core structure intact.

Even when three main CO_2_ laser parameters are coordinated to ensure the planned intensity of fibre surface modification, local heat damage may occur due to structural irregularities and defects on the Kevlar^®^ fabric surface.

To improve productivity without overheating Kevlar^®^ fibres, the laser spot size can be increased by shifting the focal plane and thus using a positive defocused beam, but serious research needs to be conducted to coordinate laser and fabric parameters to manage the nano- and microscale Kevlar^®^ fibre surface modification process on commercial roll-to-roll laser processing equipment.

Fabric surface-contaminating defects, including fibrils and broken Kevlar^®^ fibres skin fragments, are significantly reduced during defocused beam nano- and microscale fibre surface modification, which is important if subsequent formation of functional coatings or composites is intended.

Low-power continuous wave CO_2_ laser processing is a new approach to using lasers in textile practice. To develop this direction into practical use, technology needs to be built from the ground up according to the specifics of the textile material being processed and its final use, and the process needs to be integrated/adjusted within textile technology lines. To do this, we must gather knowledge and practical experience and identify areas of use, restrictions, possible benefits, and problems. The study reported in the manuscript is just the beginning of the journey, an invitation to join the process.

## Figures and Tables

**Table 1:** The 2^2^ factorial design levels.

Designation	−1	0	1	Interval
X_1_ P, W	0.46	0.86	1.26	0.4
X_2_ LS, mm/s	100	125	150	25

**Table 2:** Laser pre-treatment parameters of fabric samples.

Designation	Power, W	Laser Speed, mm/s	Raster Step, µm
V1KM2+440_laz	1.26 (+1)	150 (+1)	80
V2KM2+440_laz	0.46 (−1)	150 (+1)	80
V3KM2+440_laz	1.26 (+1)	100 (−1)	80
V4KM2+440_laz	0.46 (−1)	100 (−1)	80
V0KM2+440_laz	0.91	120	80
V2KM2600_laz	0.46	150	80
V3KM2600_laz	1.26	100	80

Shading distinguishes experimental sets of samples.

**Table 3:** Processing plan for KM2+440 and KM2600 fabric samples.

Designation	Laser Pre-Proc.	Graphene Layers		
		1k	2k	3k
KM2_440	-	-	-	-
V1KM2+440_laz	x	-	-	-
V1KM2+440_laz_1k	x	x	-	-
V1KM2+440_laz_2k	x	x	x	-
V2KM2+440_laz	x	-	-	-
V2KM2+440_laz_1k	x	x	-	-
V2KM2+440_laz_2k	x	x	x	-
V3KM2+440_laz	x	-	-	-
V3KM2+440_laz_1k	x	x	-	-
V3KM2+440_laz_2k	x	x	x	-
V4KM2+440_laz	x	-	-	-
V4KM2+440_laz_1k	x	x	-	-
V4KM2+440_laz_2k	x	x	x	-
V0KM2+440_laz	x	-	-	-
V0KM2+440_laz_1k	x	x	-	-
V0KM2+440_laz_2k	x	x	x	-
KM2600	-	-	-	-
KM2600_1k	-	x	-	-
KM2600_2k	-	x	x	-
KM2600_3k	-	x	x	x
V2KM2600_laz	x	-	-	-
V2KM2600_laz_1k	x	x	-	-
V2KM2600_laz_2k	x	x	x	-
V3KM2600_laz	x	-	-	-
V3KM2600_laz_1k	x	x	-	-
V3KM2600_laz_2k	x	x	x	-

Shading distinguishes experimental sets of samples.

**Table 4:** Processed KM2+440 fabric samples: junction rupture force and tensile strain.

Designation	Junction Rupture Force JRF, N		Tensile Strain, %		JRF Increase, %
	Mean	±	Mean	±	
KM2+440	2.42	0.04	2.37	0.13	
V1KM2+440_laz	3.65	0.05	2.10	0.07	51%
V1KM2+440_laz_1k	5.87	0.10	2.32	0.08	143%
V1KM2+440_laz_2k	6.36	0.16	2.57	0.19	163%
V2KM2+440_laz	4.82	0.08	2.74	0.11	99%
V2KM2+440_laz_1k	6.85	0.04	2.90	0.14	183%
V2KM2+440_laz_2k	10.23	0.23	3.92	0.10	323%
V3KM2+440_laz	4.68	0.05	2.74	0.12	93%
V3KM2+440_laz_1k	5.60	0.15	2.79	0.08	132%
V3KM2+440_laz_2k	7.84	0.19	3.11	0.07	224%
V4KM2+440_laz	4.10	0.07	2.43	0.10	70%
V4KM2+440_laz_1k	4.25	0.08	2.85	0.21	76%
V4KM2+440_laz_2k	6.64	0.09	3.35	0.17	175%
V0KM2+440_laz	3.18	0.04	2.00	0.11	32%
V0KM2+440_laz_1k	5.90	0.12	2.35	0.07	144%
V0KM2+440_laz_2k	6.73	0.07	2.90	0.11	178%

“±” Confidence interval, confidence level 95%; Shading distinguishes experimental sets of samples.

**Table 5:** Processed KM2600 fabric samples: junction rupture force and tensile strain values.

Designation	Junction Rupture Force JRF, N		Tensile Strain, %		JRF Increase
	Mean	±	Mean	±	
KM2600	5.33	0.33	4.37	0.36	
KM2600_1k	7.92	0.29	3.14	0.25	49%
KM2600_2k	9.12	0.26	4.25	0.20	71%
KM2600_3k	9.62	0.32	2.99	0.19	81%
V2KM2600_laz	6.79	0.16	3.59	0.18	27%
V2KM2600_laz_1k	8.30	0.41	3.91	0.14	56%
V2KM2600_laz_2k	10.13	0.44	4.21	0.23	90%
V3KM2600_laz	6.29	0.18	3.81	0.23	18%
V3KM2600_laz_1k	7.20	0.34	4.30	0.17	35%
V3KM2600_laz_2k	8.14	0.34	3.58	0.17	53%

“±” Confidence interval, confidence level 95%; Shading distinguishes experimental sets of samples.

## Data Availability

The original contributions presented in this study are included in this article. Further inquiries can be directed to the corresponding author.
